# Comprehensive characterization of the epigenetic landscape in Multiple Myeloma

**DOI:** 10.7150/thno.54453

**Published:** 2022-01-16

**Authors:** Elina Alaterre, Sara Ovejero, Laurie Herviou, Hugues de Boussac, Giorgio Papadopoulos, Marta Kulis, Stéphanie Boireau, Nicolas Robert, Guilhem Requirand, Angélique Bruyer, Guillaume Cartron, Laure Vincent, Anne Marie Martinez, José Ignacio Martin-Subero, Giacomo Cavalli, Jerome Moreaux

**Affiliations:** 1 Department of Biological Hematology, CHU Montpellier, Montpellier, France.; 2 Institute of Human Genetics, UMR 9002 CNRS-UM, Montpellier, France.; 3 Diag2Tec, Montpellier, France.; 4 Institut d'Investigacions Biomèdiques August Pi i Sunyer (IDIBAPS), Barcelona, Spain.; 5 Centro de Investigación Biomédica en Red de Cáncer, CIBERONC, Madrid, Spain.; 6 Clinical Hematology. CHU Montpellier, Montpellier, France.; 7 University of Montpellier, UFR Medicine, Montpellier, France.; 8 UMR-CNRS 5535, Institut de Génétique Moléculaire de Montpellier, 34090 Montpellier, France.; 9 Departament de Fonaments Clinics, Facultat de Medicina, Universitat de Barcelona, Barcelona, Spain.; 10 Institució Catalana de Recerca i Estudis Avançats (ICREA), Barcelona, Spain.; 11 Institut Universitaire de France (IUF).

**Keywords:** Epigenetics, Multiple myeloma, Histone modifications, Therapeutic target, Precision medicine

## Abstract

**Background:** Human multiple myeloma (MM) cell lines (HMCLs) have been widely used to understand the molecular processes that drive MM biology. Epigenetic modifications are involved in MM development, progression, and drug resistance. A comprehensive characterization of the epigenetic landscape of MM would advance our understanding of MM pathophysiology and may attempt to identify new therapeutic targets.

**Methods:** We performed chromatin immunoprecipitation sequencing to analyze histone mark changes (H3K4me1, H3K4me3, H3K9me3, H3K27ac, H3K27me3 and H3K36me3) on 16 HMCLs.

**Results:** Differential analysis of histone modification profiles highlighted links between histone modifications and cytogenetic abnormalities or recurrent mutations. Using histone modifications associated to enhancer regions, we identified super-enhancers (SE) associated with genes involved in MM biology. We also identified promoters of genes enriched in H3K9me3 and H3K27me3 repressive marks associated to potential tumor suppressor functions. The prognostic value of genes associated with repressive domains and SE was used to build two distinct scores identifying high-risk MM patients in two independent cohorts (CoMMpass cohort; n = 674 and Montpellier cohort; n = 69). Finally, we explored H3K4me3 marks comparing drug-resistant and -sensitive HMCLs to identify regions involved in drug resistance. From these data, we developed epigenetic biomarkers based on the H3K4me3 modification predicting MM cell response to lenalidomide and histone deacetylase inhibitors (HDACi).

**Conclusions:** The epigenetic landscape of MM cells represents a unique resource for future biological studies. Furthermore, risk-scores based on SE and repressive regions together with epigenetic biomarkers of drug response could represent new tools for precision medicine in MM.

## Introduction

Multiple myeloma (MM) is a B cell neoplasia characterized by the accumulation of clonal plasma cells in the bone marrow. Despite the survival improvement provided by current treatments, the majority of patients relapse and eventually become resistant to all treatments. The median overall survival of MM patients is 6 years 1. MM is characterized by a high degree of biological, genetic and intra-clonal heterogeneity 2,3. The development of MM is usually accompanied by a series of genetic alterations, such as cytogenetic abnormalities, primary and secondary chromosomal translocations and oncogenic activation. Identifying these alterations is highly valuable in understanding the pathogenesis of MM and predicting outcome and therapeutic response. Chromosomal alterations, including t(4;14) translocation, 17p13 deletion and 1q21 gain correlate with poor disease prognosis 4. Several mutations also negatively impact on survival, including mutations in *CCND1* and genes involved in DNA repair pathways (*TP53*, *ATM*, *ATR* and *ZNFHX4*) 5.

The epigenetic control of gene expression plays an essential role in the regulation of cell fate and cell identity. Aberrant changes in key regulatory chromatin features, such as DNA methylation and histone post-translational modifications (PTMs), are involved in MM pathophysiology and drug resistance 6. The histone PTM landscape is particularly dynamic and constantly evolving 7. Structural changes in active euchromatin or silenced heterochromatin are controlled by epigenetic enzyme complexes of chromatin writers, readers and erasers. Histone deacetylases (HDAC) are dysregulated in MM with an aberrant overexpression of class I HDACs in a subset of patients, which is associated with a shorter overall survival 8. HDAC inhibitors (HDACi) are now used in the treatment of several hematologic malignancies, including MM 9. Moreover, epigenetic biomarkers for MM can be used as predictive and prognostic indicators to guide diagnosis and treatment 10-13. These data suggest that individualizing therapy based on epigenetic biomarkers has the potential to increase therapeutic efficacy for MM patients.

Human MM cell lines (HMCLs) have been widely used for the understanding of MM biology, identifying target genes, screening of anti-myeloma drugs and, more recently, characterizing the MM mutational landscape 14,15. In the past few years, we have derived a large cohort of patient-derived HMCLs that remain dependent on the addition of exogenous MM growth factors, reflecting primary tumor conditions 16. Using these myeloma cell lines as well as commercial myeloma cell lines, we described their molecular diversity by analyzing their gene expression profile and mutational landscape and showed that HMCL molecular diversity reflects part of the molecular heterogeneity of primary MM cells 14,16. However, the global epigenetic landscape of HMCLs has never been described.

A comprehensive characterization of the epigenetic landscape of HMCLs would advance our understanding of MM pathophysiology and its analysis might lead to the identification of new therapeutic targets for future application in MM. In this study, we present the first epigenetic landscape of HMCLs. We generated chromatin immunoprecipitation sequencing (ChIP-seq) maps for a set of active (H3K4me1, H3K4me3, H3K27ac, H3K36me3) and inactive (H3K9me3, H3K27me3) histone PTMs on 16 HMCLs, representative of the molecular heterogeneity of MM 14,16. Differential analysis of PTM profiles on HMCLs highlighted links between histone modifications and cytogenetic abnormalities. We have identified super-enhancers (SE) associated with genes involved in the biology of MM, such as *MAF*, *MYC*, *CCND1*, *CCND2*, *TRAF3* or* NSD2*, and repressive domains, characterized by the co-localization of the H3K9me3 and H3K27me3 modifications on promoters of genes with potential tumor suppressor function. We used genes belonging to these two subsets and associated with prognostic value to build two distinct scores that can predict MM patient survival. Finally, we have identified H3K4me3 active promoters associated with the sensitivity of HMCL cells to lenalidomide and the HDACi romidepsin. From these data, we have developed epigenetic biomarkers based on this H3K4me3 PTM predicting the response to lenalidomide and romidepsin HDACi. Together, these data constitute a rich source of information that might guide analyses towards the identification of novel biomarkers and therapeutic targets for MM.

## Methods

### Samples

XG human myeloma cell lines were obtained as previously described 16. AMO-1, OPM2 and SKMM2 were purchased from DSMZ (Braunschweig, Germany) and RPMI8226 from ATCC (Manassas, USA). JJN3 was kindly provided by Dr.Van Riet (Brussels, Belgium), and KMS-12-BM by Dr Otsuki (Okayama, Japan). HMCLs characteristics are available in Table 1.

Bone marrow samples were collected after patient's written informed consent in accordance with the Declaration of Helsinki and institutional research board approval from Montpellier University Hospital. Bone marrow was collected from 69 patients at diagnosis and 28 patients at relapse, this cohort was called Montpellier cohort. MM cells of patients were purified using anti-CD138 MACS microbeads (Miltenyi Biotec, Bergisch Gladbach, Germany). We also used RNA sequencing data of 674 newly diagnosed MM patients with longitudinal follow-up from the Multiple Myeloma Research Foundation CoMMpass trial (NCT01454297; version IA11a), termed in the following CoMMpass cohort.

Normal plasma cells were generated using a 3-step *in vitro* model starting from purified memory B cells from 3 different healthy donors as reported 17,18.

### HMCLs response to drug treatment

HMCLs were cultured in RPMI-1640 medium (Gibco, Thermo Fisher Scientific, Waltham, USA) supplemented with 10% fetal bovine serum (Eurobio, Les Ulis, France) and IL-6 (Peprotech, Rocky Hill, USA) for XG cell lines. We evaluated the sensitivity of HMCLs to GSK525762, Chaetocin (Sigma-Aldrich, Saint-Louis, USA), EPZ-6438, lenalidomide and romidepsin (Selleckchem, Houston, USA). The IC50 of GSK525762, Chaetocine, lenalidomide and romidepsin was determined at day 4 using the CellTiter-Glo assay (Promega, Madison, USA), as previously described 11,19. HMCLs were cultured 8 days with or without EPZ-6438. Cell concentration and viability were assessed using trypan blue dye exclusion test, as previously described 12. The data represent the mean ± standard deviation of three independent experiments that were carried out on sextuplet culture wells (Figure 6A and Supplementary Figure 16A).

### Gene expression profiling and statistical analysis

HMCLs, patients MM cells and normal plasma cells RNA sequencing was done as previously described 12. The RNA sequencing (RNA-seq) library preparation was done with 150 ng of input RNA using the Illumina TruSeq Stranded mRNA Library Prep Kit. Paired-end RNA-seq were performed with Illumina NextSeq sequencing instrument (Helixio, Clermont-Ferrand, France). RNA-seq read pairs were mapped to the reference human GRCh37 genome using the STAR aligner 20. All statistical analyses were performed with the statistics software R (version 3.6.2; https://www.r-project.org), and R packages developed by Bioconductor project (https://www.bioconductor.org) 21. The expression level of each gene was summarized and normalized using the DESseq R/Bioconductor package 22.

For the Montpellier cohort and HMCLs, Affymetrix U133P chips were also used, as previously described 23,24 (ArrayExpress public database under accession numbers E-TABM-937 and E-TABM-1088), to calculate previously published risk scores including RS score 25, HRS score 26, IFM score 27, EZ score 12 and HA score 11.

### Whole Exome sequencing (WES) and variant calling

WES of human MM cell line was performed as described above 14. WES public data were used for KMS-12-BM 28. Reads were mapped to the reference human hg19 using the Bowtie 2 aligner version 2.3.2 29. SAMtools version 1.5 was used to convert SAM files to sorted BAM files 30. Indel realignment, base quality recalibration and variant calling steps were completed with GATK 3.8-1 31. ANNOVAR was used to annotate variants 32. Then, filters described in Vikova *et al.* were applicated 14.

### HMCLs ChIP-seq

HMCLs were cross-linked in formaldehyde at a final concentration of 1% for 8 minutes. All experiments reagents were included in the iDeal ChIP-seq kit for histones (Diagenode, Liege, Belgium). Sonication was performed using a Bioruptor Plus sonication device (Diagenode, Liege, Belgium) under the optimal conditions to shear cross-linked DNA to fragments of 100-300 base pairs in length. ChIP were conducted with the IPStar Compact Automated System (Diagenode) and the iDeal ChIP-seq kit for histones (Diagenode, C01010059). ChIP were performed starting from 1,000,000 cells. Crossed-linked DNA was incubated 13h with H3K4me1 (Diagenode, C15410194), H3K4me3 (Diagenode, C15410003), H3K9me3 (Diagenode, C15410193), H3K27ac (Diagenode, C15410196), H3K27me3 (Diagenode, C15410195) or H3K36me3 (Diagenode, C15410192) antibody and 3h with the beads. After 5 min washes, eluates were recovered and reverse cross-linked for 4h at 65°C. Samples were treated for 1h with RNase at 37°C, prior to DNA purification with the Auto IPure kit v2 (Diagenode, C03010010). Libraries were performed using NEBNext Ultra II DNA Library Prep Kit for Illumina (New England Biolabs). ChIP-seq were performed with Illumina NextSeq500 technology (Helixio, Clermont-Ferrand, France) using the following parameters: single-ended, 50bp, 40 million reads.

ChIP-seq for H3K4me1, H3K4me3, H3K9me3, H3K27ac, H3K27me3 and H3K36me3 histone marks of 4 MM patients were performed using purified MM cells (MMC) of 4 MM patients as previously described 33. LiftOver function from the rtracklayer R package (version 1.52.1) was used to change the genomic coordinate of MMC peaks, from hg38 to hg19. Peaks identified in MMC were compared to HMCL peaks for each histone marks using the findOverlaps function from the regioneR R package (version 1.24.0).

Reads were mapped to the human reference genome (hg19) using Bowtie2 (version 2.3.2) 29. Peak-calling was performed using MACS2 (version 2.1.2) 34. Peak annotation and differential binding analyses of ChIP-seq peak data were performed using ChIPseeker 35 and DiffBind 36 R/bioconductor packages, respectively. Heatmaps and average profiles were generated by deeptools (version 3.5.0) 37. Super-enhancers (SE) were identified using the ROSE (Rank Ordering of Super-Enhancers) algorithm 38,39 based on H3K27ac and H3K4me1 ChIP-seq signals. TSS exclusion zone size was adjusted to 2500 bp to exclude promoter regions. Transcriptionally active genes were assigned to super-enhancers using a simple proximity rule (50 kb window). Scores were built using our previously published methodology 10. Genes composing super-enhancer and repressive domain scores were selected for their prognostic significance using Maxstat R function and the Benjamini Hochberg multiple testing correction. Score values correspond to the sum of the Cox beta-coefficients of each gene, weighted by ± 1 if patient MM cells signal for the gene of interest is above or below the Maxstat reference value of this gene. The statistical significance of differences in overall survival between patients' groups was calculated using the log-rank test and survival curves were plotted using the Kaplan-Meier method. CoMMpass and Montpellier cohorts were used as training and validation cohorts, respectively. Statistical differentially bound regions between drug-sensitive and -resistant HMCLs were identified with DiffBind R/bioconductor package (FDR ≤ 0.05).

### Generation of HMCL stably expressing isoform 1 Cul4B-GFP

XG1 cells were infected with lentiviral vectors containing GFP (PS100093V, OriGene) or GFP-tagged Cul4B isoform 1 (Cul4B-GFP) (RC206935L4V, OriGene). The cell lines were selected with puromycin and sorted by FACS based on GFP expression levels.

## Results

### Epigenetic landscape of HMCLs

**Figure 1:**
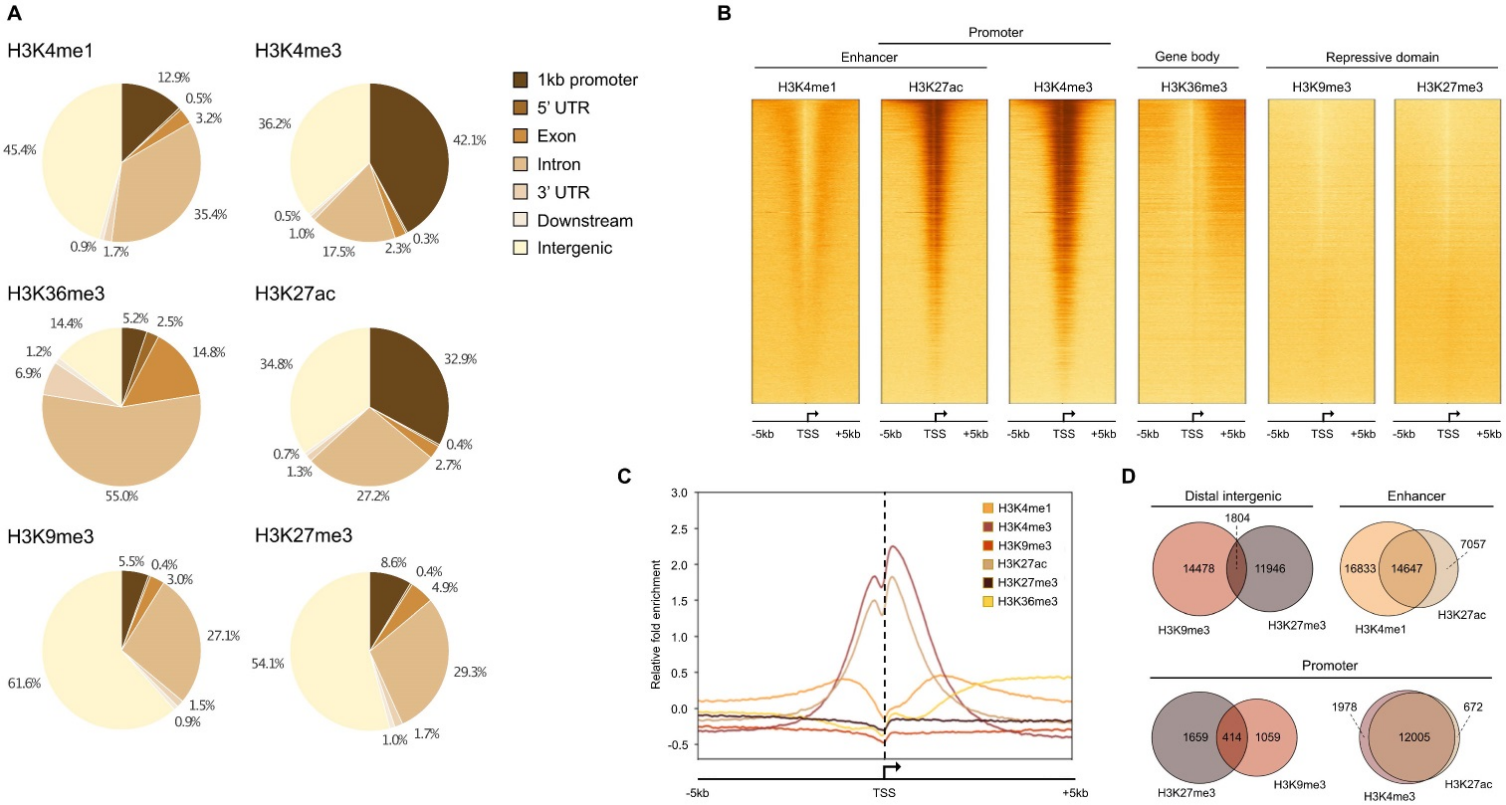
Histone mark enrichment distribution in HMCLs. (A) Pie chart showing the distribution of H3K4me1, H3K4me3, H3K9me3, H3K27ac, H3K27me3 and H3K36me3 peaks in promoter, 5' UTR, exon, intron, 3' UTR, downstream and distal intergenic regions. The peaks were identified by MACS and were annotated using ChIPseeker R package. Results are expressed as the mean percentage distribution of the 16 HMCLs. (B) Heatmap of histone modification peaks binding to the TSS regions (from -5 kb to 5 kb) ranked by histone mark signal in the XG6 cell line. (C) ChIP-seq profiles across 5 kb regions centered on the TSS of the full genome of XG6 cell line. The y axis shows the histone ChIP-seq signal normalized using the log2 ratio (histone *vs* input) normalization. Heatmaps and average profiles of histone mark enrichment were generated by deeptools using all known genes for each individual histone modification. (D) Venn diagram of overlapped region between H3K9me3 and H3K27me3 in intergenic regions and promoters, H3K4me1 and H3K27ac in enhancer regions, and H3K4me1 and H3K27ac in promoters in XG6.

We performed ChIP-seq analysis using 16 HMCLs characterized by molecular and genetic variability, which reflect part of the heterogeneity of the disease (Table 1) 14,16. To evaluate the epigenetic landscape of these HMCLs, two repressive marks (H3K9me3 and H3K27me3) and four active marks (H3K4me1, H3K4me3, H3K27ac and H3K36me3) were selected. The six histone marks used in this study are those proposed by the International Human Epigenome Consortium as the most informative ones and constitute an essential part of the reference epigenome (http://ihec-epigenomes.org/research/reference-epigenome-standards/). Specifically, H3K36me3 is related to transcriptional elongation in the gene body, H3K4me3 to poised/active promoters, H3K4me1 to poised/active enhancers, H3K27ac to determine active regulatory elements (both enhancers and promoters), H3K9me3 to detect heterochromatin and H3K27me3 to detect Polycomb-repressed chromatin. The annotation of the peaks reveals that the enhancer-related H3K4me1 modification is mainly found in distal intergenic (45.4% ±2.6%) and intron (35.4% ±2.4%) regions whereas H3K27ac, a modification known to be associated with both active enhancers and promoters, is mainly found in distal intergenic (34.8% ±4.0%) and promoter (32.9% ±4.6%) regions (Figure 1A). H3K4me3 is mainly located at promoters, as expected for a mark of actively transcribed gene promoters, but also in intergenic regions (promoters: 42.1% ±6.8%; intergenic: 36.2% ±5.0%). The H3K4me3 and H3K27ac marks exhibit the same profile surrounding the transcription start site (TSS) with a major peak located downstream the TSS (Figure 1B-D). The H3K4me1 mark is located both upstream and downstream to the TSS (Figure 1B-D). On enhancer regions, the majority of H3K27ac peaks overlapped with H3K4me1 peaks, revealing active enhancers, whereas non-overlapping H3K4me1 peaks were associated with poised enhancers (Figure 1D) 40. The H3K36me3 modification is identified downstream to the TSS, on the body of active genes with a distribution of intron and exon regions representing 69.8% of total regions. The repressive marks, H3K9me3 and H3K27me3, respectively detecting HP1- and Polycomb-repressed chromatin, were essentially found in intergenic regions (61.6% ±6.2% and 54.1% ±4.3%, respectively), with distinct localization, but also on gene promoters (Figure 1A-D). A small fraction of these promoters was enriched by H3K9me3 and H3K27me3 co-localization. Moreover, we compared our results with the ChIP-seq data of 4 MM patients 33. We identified that more than 80% of MM patients peaks were commonly found in HMCL data for H3K4me1, H3K4me3, H3K9me3, H3K27me3 and H3K36me3 histones marks. Concerning H3K27ac, 70% of overlap was identified comparing HMCLs and primary MMC from patients (Supplementary Table 1).

**Figure 2:**
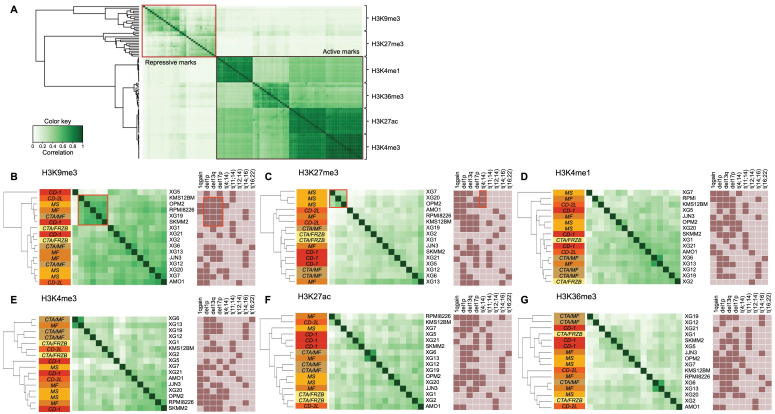
Differential histone enrichment evaluation in 16 HMCLs. Correlation heatmaps of H3K4me1, H3K4me3, H3K9me3, H3K27ac, H3K27me3 and H3K36me3 marks were generated using Diffbind R package. A) Correlation heatmap, using occupancy data (peaks identified by MACS), performed on the 6 histone marks in HMCLs shows distinct separation from each other and between active and repressive histone marks. B) Correlation heatmap, using affinity data (read count), highlights the link between histone modifications and both HMCLs molecular classification and cytogenetic abnormalities (dark box: presence, light box: absence). The clustering of the samples was calculated using the cross-correlations of each row of the binding matrix. The correlations range from 0 (no correlation, white) to 1 (strong correlation, dark green). Orange boxes encompass interest clusters identified using unsupervised hierarchical clustering. CD-1, CD-2L, CTA/MF, CTA/FRZB, MF and MS correspond to the transcriptional classification of HMCLs.^16^

Figure 2A shows the global profile of histone modifications on the 16 HMCLs. The clustering based on peak localizations distinguishes each mark from all others and repressive (H3K9me3 and H3K27me3) from active (H3K4me1, H3K4me3, H3K27ac and H3K36me3) marks. TSSs of actively transcribed genes are marked by H3K4me3 and H3K27ac modifications, which explains the high similarity between H3K4me3 and H3K27ac profiles (Figure 1C and 1D) 40. When each mark is observed independently, the clustering of HMCLs reveals subgroups associated to different cytogenetic abnormalities or HMCLs transcriptional classification 16. Differential analysis on H3K9me3 reveals a highly similar cluster, composed of KSM-12-BM, OPM2, RPMI8226, XG19 and SKMM2, characterized by the association of del1p, del13q and del17p cytogenetic abnormalities (Figure 2B and Supplementary Table 2) (r > 0.5). The H3K27me3 analysis distinguishes the group of HMCLs harboring the t(4;14) translocation (XG7, XG20 and OPM2) from other HMCLs (Figure 2C and Supplementary Table 3) (r > 0.5). The t(4;14) translocation results in *IgH*/*MMSET* hybrid transcripts inducing overexpression of the *MMSET* gene 41. There is a correlation between the number of H3K27me3 peaks and *MMSET* mRNA expression in the groups of HMCLs without t(4;14) translocation (R^2^ = 0.33; *P*-value < 0.02) (Supplementary Figure 1A) whereas no correlation is observed in t(4;14) cell lines (data not shown). However, the percentage of reads in H3K27me3 peaks is correlated to the *MMSET* mRNA expression in all HMCLs (R^2^ = 0.48; *P*-value < 0.01) (Supplementary Figure 1B). These results suggest that H3K27me3 localizations are very similar in t(4;14) HMCLs and that the global enrichment of H3K27me3 increases with the deregulation of *MMSET* without increasing the total number of H3K27me3 sites on the genome. Popovic *et al.* showed that *MMSET* overexpression led to a global loss of H3K27me3 and an enrichment of this modification on specific loci 42. Overexpression of MMSET is associated with a shift in the genomic localization of the H3K27 methyltransferase EZH2, imposing EZH2 and H3K27me3 accumulation at specific loci 43. Differentially bound site analysis revealed around 46000 sites significantly differentially associated to the H3K27me3 modification in t(4;14) HMCLs (MMSET subgroups) compared to HMCLs without t(4;14) translocation (FDR ≤ 0.05) (Supplementary Figure 2A). Of these, 1822 sites are located on gene promoters, with 809 gene promoters presenting lower H3K27me3 levels *versus* 1013 gene promoters enriched in H3K27me3 in the MMSET subgroup (FDR ≤ 0.05) (Supplementary Figure 2B and 2C). Among this set of genes, we found genes involved in the induction of apoptosis (*TP73* and *BNIP3*) and the cell cycle regulation (*CEND1*, *CCNO*, *CDKN1C* and *CDK2AP1*). Comparing gene expression profiles of the 3 HMCLs harboring t(4;14) translocation (XG7, XG20 and OPM2) with HMCLs without the translocation revealed 144 genes significantly overexpressed and 23 genes significantly downregulated in the t(4;14) HMCLs. Among the 144 genes overexpressed in t(4;14) HMCLs, the level of H3K27me3 is significantly lower on the promoter of 31 genes in t(4;14) HMCLs compared to HMCLs without t(4;14) translocation. The promoter of 3 genes is significantly enriched in H3K27me3 in HMCLs harboring a t(4;14) translocation among the 23 genes downregulated (Supplementary Figure 2D and Supplementary Table 4). Global analyses of H3K4me1, H3K4me3, H3K27ac and H3K36me3 modifications do not provide a clear link between cytogenetic abnormalities and histone modifications, while H3K27ac and H3K36me3 clustering tends to group HMCLs by HMCL molecular subgroups (Figures 2E-G). We also studied the link between histone modification profiles and frequently mutated genes in HMCLs (Supplementary Figure 3). Interestingly, HMCLs with *TP53* bi-allelic events are characterized by a specific H3K9me3 profile (Supplementary Figure 4).

### SE-risk score predicting survival in MM patients

**Figure 3:**
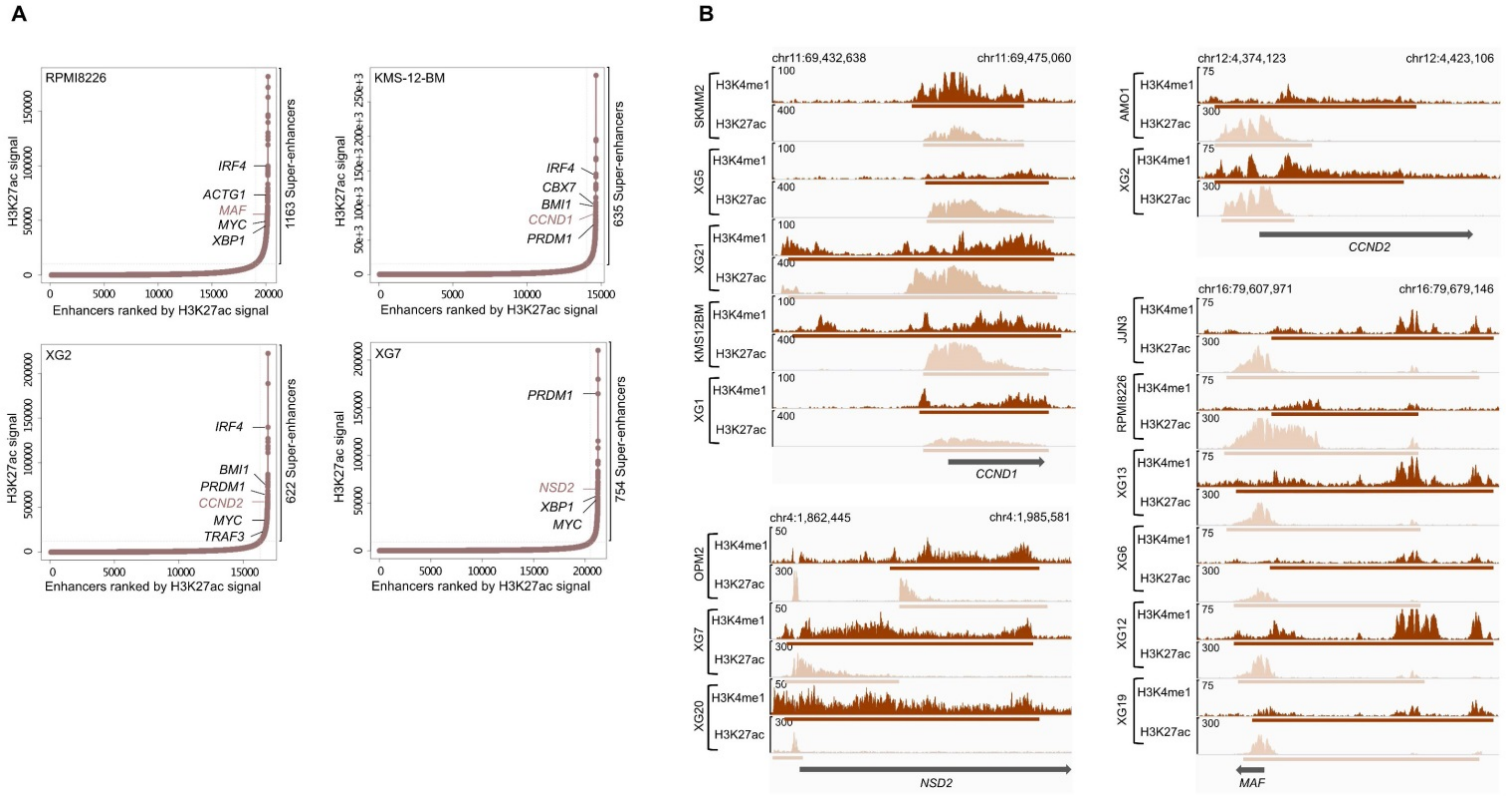
Super-enhancers identified in HMCLs. (A) Total of H3K27ac ChIP-seq signal in units of reads per bin mapped reads in enhancer regions for all enhancers in RPMI8226, KMS-12-BM, XG2 and XG7 cell lines harboring translocation which targets *MAF*, *CCND1*, *CCND2* and *NSD2* gene, respectively. Enhancers are ranked by increasing H3K27ac ChIP-seq signal. (B) Gene tracks of H3H4me1 and H3K27ac ChIP-seq occupancy at super-enhancers near genes involved in MM pathogenesis. Super-enhancers of *CCND1*, *NSD2* and *CCND2* overlap the TSS region while *MAF* enhancer is localized downstream of the gene. The x axis shows the genomic position and H3K27ac and H3K4me1 enhancers-containing regions are depicted with a beige and brown box, respectively. The y axis shows signal coverage of ChIP-seq occupancy in units of reads per bin mapped reads.

H3K4me1 and H3K27ac modifications are known to be associated with enhancer regions and, more specifically, active enhancers can be identified by co-occupancy of H3K4me1 and H3K27ac 40. Super-enhancers consist of a set of large enhancer domains displaying physical proximity (+/- 12.5kb) and are associated with genes that control and define cell identity 38,39. Using the MM1S cell line, Lovén et al. identified super-enhancers associated with key MM genes, including MYC, IRF4, PRDM1, XBP1, CCND2, PIM1, BCL-xL and MCL1 39.We identified a number ranging from 607 to 2510 predicted super-enhancers in each of the HMCLs (Supplementary Table 5). These super-enhancers differed from typical enhancers in both size and H3K4me1 and H3K27ac levels (Figure 3A). Enhancers tend to loop to and associate with adjacent genes in order to activate their transcription 44. Using a simple proximity rule, we assigned all TSSs to super-enhancers within a 50 kb window 45. Among the SE-associated genes identified, we found genes identified in the study of Lovén et al. (*MYC*, *IRF4*, *PRDM1*, *XBP1* and *CCND2*) but also other key MM genes, such as *ACTG1*, *MAF*, *CCND1*, *TRAF3* and *NSD2* (*MMSET*). Among them, we found SE-associated genes targeted by MM translocations (Table 1 and Figure 3B).

**Figure 4:**
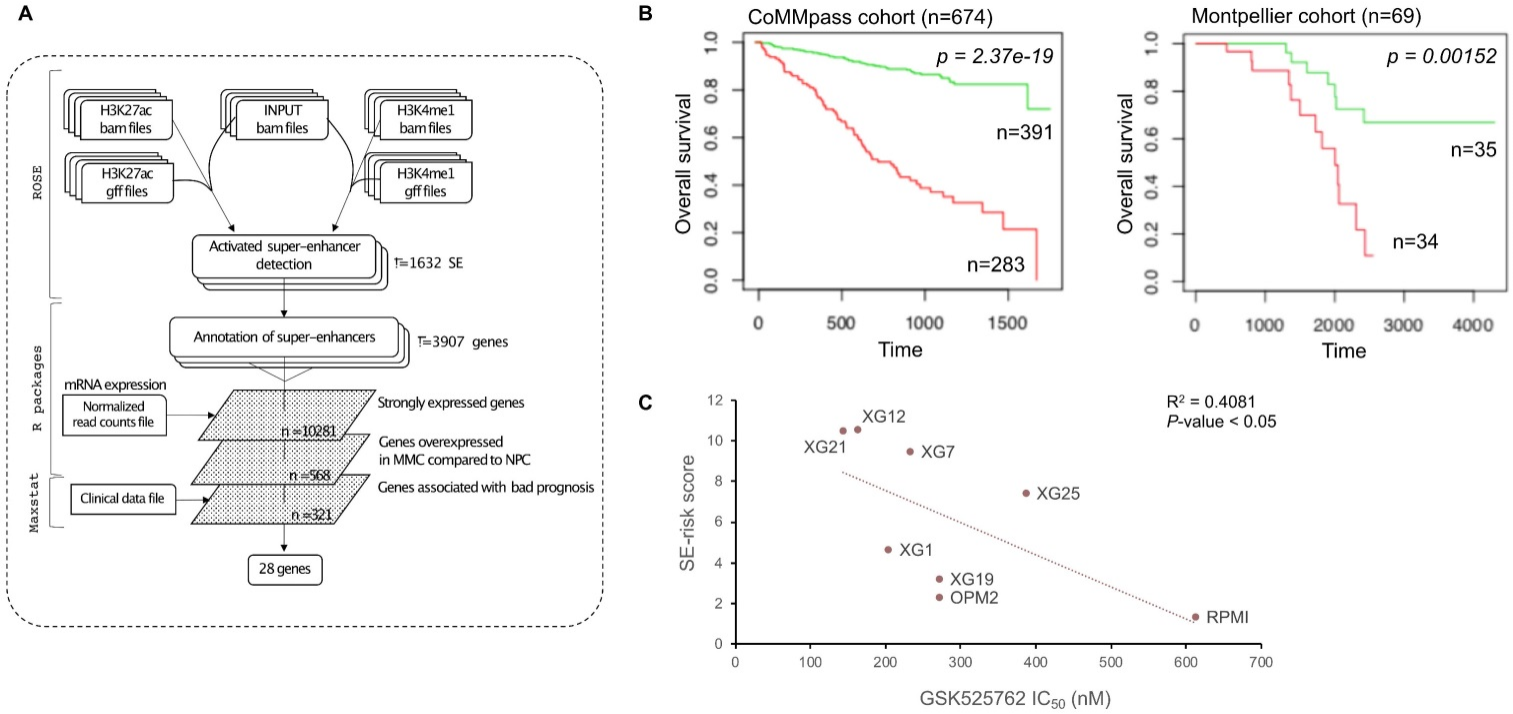
SE-risk score can predict survival in MM patients. (A) SE-risk score pipeline using first ROSE algorithm on ChIP-seq data of HMCLs to find super-enhancers. SE-associated genes are identified using RNA-seq of HMCLs. Genes of interest are then filtered according to their expression in HMCLs, plasma cells of MM patients (MMC) and normal plasma cells (NPC), and their prognostic value in MM patients. (B) Prognostic value of the SE-risk score in MM. Patients of the CoMMpass cohort (n = 674) were ranked according to the increased SE-risk score and a maximum difference in OS was obtained with SE-risk score of -4.0 splitting patients into high-risk (n = 283; red curve) and low-risk (n = 391; green curve) groups. SE-risk score also had a prognostic value in an independent cohort of 69 patients (Montpellier cohort). To compute the SE-risk score in Montpellier cohort, the proportion of patients of the CoMMpass cohort for each gene was applied on the Montpellier cohort to determine gene cut-point. The same gene beta-coefficient and score cut-point was applied to distinguish high-risk (n = 34: red curve) from low-risk (n = 35; green curve) groups. (C) SE-associated gene score correlates with the response to GSK525762 in HMCLs. Linear regression analysis of the SE-associated gene score in function of the IC50 of GSK525762 in 8 HMCLs. Coefficient of determination R^2^ represents the square of the Pearson correlation coefficient (r) (Pearson correlation test).

We used these SE-associated genes to build a score predicting survival in MM patients. First, we selected SE-associated genes with strong expression in HMCLs, overexpressed in plasma cells of MM patients compared to normal plasma cells and associated with shorter overall survival in MM patients using the Maxstat algorithm 46 (Figure 4A). Twenty-eight genes fulfilled these criteria: *BSG* (CD147), *HK2*, *HNRNPC*, *HSPA9*, *IL10*, *ILF3*, *LDHB*, *MDH1*, *MYBPC2*, *NCL*, *NUDC*, *PARP1*, *PDIA6*, *PRPS1*, *RPL8*, *RPL13A*, *RPL27A*, *RPL35*, *SF3B2*, *SLC7A5*, *SLC25A39*, *SMARCA4*, *SPN* (CD43), *STC2*, *THY1* (CD90), *TNPO2*, *TPR* and *YWHAQ* (Supplementary Figures 5 and 6). Then, the prognostic information of these 28 SE-associated genes was combined to build a SE-risk score. This score is defined by the sum of the beta coefficients of the Cox model for each prognostic gene, weighted by ±1 if the patient plasma cells signal for a given gene is above or below the Maxstat reference value of this gene as previously described 47. Using two independent cohorts, the SE-risk score had a prognostic value when used to split patients into two groups using the Maxtstat R function. The score splits patients into a high-risk group (score > -4.0) and a low-risk group (score < -4.0) in the CoMMpass (*P*-value < 0.0001; high-risk group: 58.0% of patients; low-risk group: 42.0% of patients) and Montpellier (*P*-value < 0.005; high-risk group: 50.7% of patients; low-risk group: 49.3% of patients) cohorts (Figure 4B). Investigating the link between the SE-risk score and cytogenetic abnormalities, SE-risk score values were significantly higher in MM patients with del(13q), del(17p), del(1p), 1q gain and t(12;14) in the CoMMpass cohort (Supplementary Figure 7). We evaluated the evolution of the score from diagnosis to relapse in 14 MM patients with paired samples. No significant difference between the two groups was identified (Supplementary Figure 8). However, 2 MM patients presented a striking increase in SE-risk score from diagnosis to relapse. We next investigated the SE-risk score value distribution according to Affymetrix GEP-based risk scores previously reported in MM. The SE-risk score values were significantly higher in high-risk patients defined by RS score 25, and IFM score 27. The SE-risk score values were also significantly higher in intermediate and high-risk patients compared to low-risk patients defined by the International Staging System (ISS) 48. Furthermore, high-risk patients defined with the SE-risk score demonstrated a significant increase in the percentage of proliferating MM cells determined by BrdU incorporation 49 (median = 1.55%, range: 0 - 17.3%) compared to the low group (median = 0.7%; range: 0 - 7.3%) (Supplementary Figure 9). Bromodomain and extra-terminal (BET) inhibitors have been shown to repress super-enhancer-associated transcription. To evaluate the link between the score and the response of MM cells to bromodomain and extra-terminal (BET) inhibitors, we calculated the SE-associated gene score in HMCLs. Interestingly, we found a significant anti-correlation between the score and the response to a BET inhibitor in clinical development in hematological cancers (GSK525762) in HMCLs (R^2^ = 0.4081; *P*-value < 0.05) (Figure 4C). The same tendency was obtained using JQ1 and OTX015 BET inhibitors (Data not shown). Using other scores based on gene expression profiling (EZ 12, HA 11 and HRS 26 scores) calculated in 8 HMCLs, we did not find a significant correlation between these scores and the BET inhibitor sensitivity (Supplementary Figure 10). Nevertheless, a correlation was found between HRS score and the response to GSK525762 (R^2^ = 0.489; *P*-value < 0.05). High SE-associated gene score allows the identification of high-risk MM patients that could benefit from BET inhibitors. Conversely, high HRS score is associated with resistance to GSK525762 BET inhibitor.

Interestingly, 21 genes (*HK2*, *HNRNPC*, *HSPA9*, *ILF3*, *LDHB*, *MDH1*, *MYBPC2*, *NCL*, *NUDC*, *PARP1*, *PDIA6*, *PRPS1*, *RPL13A*, *RPL27A*, *SF3B2*, *SLC25A39*, *SMARCA4*, *STC2*, *TNPO2*, *TPR* and *YWHAQ*) were significantly overexpressed in HMCLs compared to plasma cells of MM patients (Supplementary Figure 5), suggesting that genes may play a role in disease progression and independence of MM cells from micro-environment. Moreover, DEMETER2 50 and CERES 51 dependency scores calculated from public datasets of RNAi 52,53 and CRISPR/Cas9-based 51 screening (Dependency Map data, Broad Institute, www.depmap.org) indicated that *YWHAQ*, *IL10*, *HK2* and *THY1* are significant essential MM genes (Supplementary Table 6 and Supplementary Figure 11). Indeed, *THY1* knockdown presented the larger DEMETER2 dependency score difference between HMCLs and all other cell types investigated. Altogether, these data show that a signature of genes associated with super-enhancers in MM could be defined allowing the identification of high-risk MM patients that could benefit from treatment with BET inhibitors.

### H3K9me3 and H3K27me3 co-localization in promoters can be associated with prognostic value in MM

**Figure 5:**
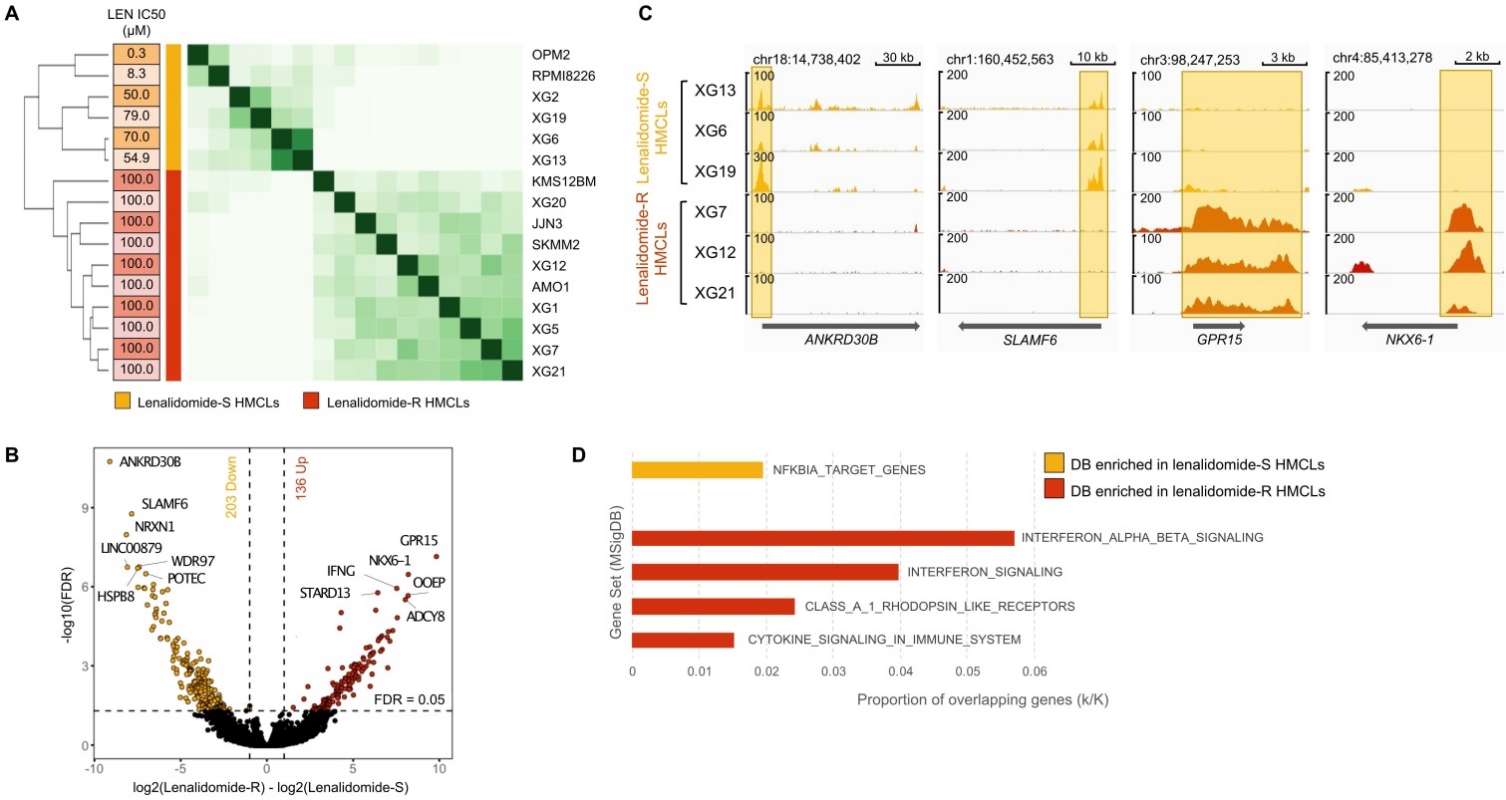
H3K9me3/H3K27me3 score can predict survival in MM patients. (A) Repressive domains characterized by co-localization of H3K9me3 and H3K27me3 enrichment on promoter of *SEMA6A* and *ARHGEF5* gene. The promoter of these genes is the most frequently enriched in H3K9me3 and H3K27me3 modifications in HMCLs. The x axis shows the genomic position and the y axis shows signal coverage of ChIP-seq occupancy in units of reads per bin mapped reads. (B) Prognostic value of the H3K9me3/H3K27me3 score in MM. Patients of the CoMMpass cohort (n = 674) were ranked according to the increased H3K9me3/H3K27me3 score and a maximum difference in OS was obtained with increased H3K9me3/H3K27me3 score of -3.45 splitting patients into high-risk (n = 390; red curve) and low-risk (n = 284; green curve) groups. High-risk group for individual gene composing H3K9me3/H3K27me3 score is associated with low gene expression (negative beta-coefficient) whereas high-risk group for H3K9me3/H3K27me3 score is associated with high score because of the multiplication of the beta-coefficient (negative value) and the weight (+1 or -1 if the signal of MM patient gene is above or below the Maxstat reference value, respectively) of genes. H3K9me3/H3K27me3 score also had a prognostic value in an independent cohort of 69 patients (Montpellier cohort). To compute the H3K9me3/H3K27me3 score in Montpellier cohort, the same method described before was used.

Several reports have demonstrated that H3K9me3 and H3K27me3 modifications are mutually exclusive in constitutive heterochromatin. It was previously suggested that H3K9me3 may crosstalk with the Polycomb mediated H3K27me3 modification to cooperate in gene repression (Figure 1D) 54-56. We decided to find gene promoters enriched by both H3K9me3 and H3K27me3 modifications to build a score based on genes associated with epigenetic transcriptional repression and identify new potential tumor suppressor genes. First, H3K9me3 and H3K27me3 overlapping regions on gene promoters were identified for all HMCLs. To identify potential tumor suppressor genes, we selected genes significantly under-expressed in plasma cells of MM patients compared to normal plasma cells, and associated them with poor outcome when their expression is low in MM cells of patients. We identified eighteen genes: *ARHGEF5*, *BIVM*, *DEF8*, *GRID2IP*, *HDAC9*, *HSPA1L*, *KDM4C*, *NLRP2*, *P4HA3*, *PAG1*, *PM20D1*, *RMND5A*, *SEMA6A*, *SFMBT2*, *THEMIS2*, *TPRKB*, *ZFP2* and *ZNF5188B* (Supplementary Figures 12 and 13). The promoters of *SEMA6A* and *ARHGEF5* genes were most frequently enriched by both H3K9me3 and H3K27me3 modifications in HMCLs (Figure 5). We combined the prognostic information from these eighteen genes into a tumor suppressor-based risk score as described above. This score splits patients into two groups in the training cohort (CoMMpass cohort, n=674,* P*-value < 0.0001): a low-risk group (score < -3.45) of 57.9% MM patients associated with global high expression of these genes and a high-risk group (score > -3.45) of 42.1% MM patients associated with global low gene expression (Figure 5B). This score also splits MM patients into low and high-risk groups (62.3% and 37.7% of patients, respectively) in the validation cohort (Montpellier cohort, n=69, *P*-value < 0.01). In the CoMMpass cohort, the score was significantly higher in non-hyperdiploid, del13q, del17p, 1q gain, t(4;14), t(12;14) and t(14;16) subgroups of patients (Supplementary Figure 14). Of the eighteen genes, thirteen genes (*ARHGEF5*, *DEF8*, *GRID2IP*, *HDAC9*, *HSPA1L*, *KDM4C*, *P4HA3*, *PAG1*, *SEMA6A*, *SFMBT2*, *THEMIS2*, *ZFP2* and *ZNF518B*) were down-regulated in HMCLs compared to primary MM cells (Supplementary Figure 12). We evaluated the link between the score and the response of MM cells to EZH2 and SUV39H1/2 inhibitors, EPZ-6438 57 and chaetocine 58, respectively. Interestingly, we found a significant anti-correlation between the score and the response to EPZ-6438 (R^2^ = 0.4348; *P*-value < 0.05) (Figure 5C) and chaetocine (R^2^ = 0.4128; *P*-value < 0.05) (Figure 5D) in HMCLs.

### H3K4me3 bound sites linked to lenalidomide and romidepsin resistance in HMCLs

Epigenetic modifications, including histone modifications, participate to MM pathogenesis and chemotherapy resistance. To better understand the epigenetic mechanisms involved in drug resistance, we explored histone marks in drug-resistant and -sensitive HMCLs. H3K4me3, a modification associated with promoters of actively transcribed genes, was chosen to identify epigenetic changes involved in drug resistance. We analyzed the relationship between the H3K4me3 landscape of HMCLs and their response to conventional drugs including bortezomib, melphalan, dexamethasone, lenalidomide and HDACi 14.

**Figure 6:**
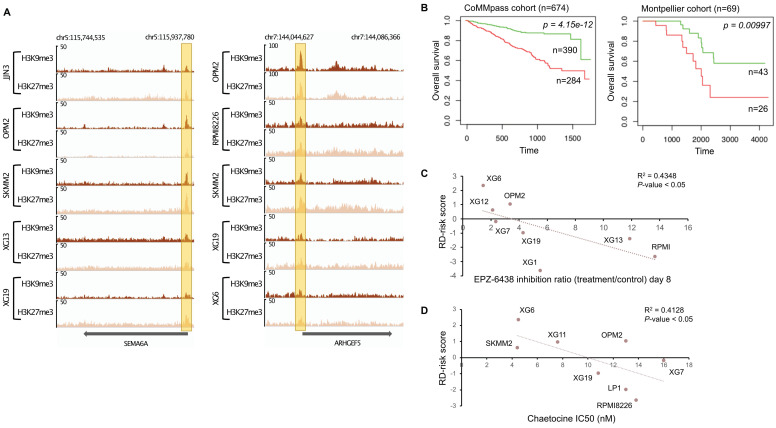
H3K4me3 modification differentially enriched in lenalidomide (LEN) -sensitive and -resistant HMCLs. We distinguished two groups of HMCLs: sensitive cell lines, the IC50 were ranged from 0.3µM to 79 µM, and resistant cell lines, the IC50 were not reached at 100µM. (A) Correlation heatmap using only the 5903 sites identified as being significantly differentially bound in lenalidomide-resistant compared to lenalidomide-sensitive HMCLs (FDR ≤ 0.05). (B) Volcano plot of differentially bound sites localized on promoters using lenalidomide-resistant *vs* lenalidomide-sensitive HMCLs contrast. Sites identified as significantly differentially bound in lenalidomide-sensitive and -resistant HMCLs are colored in orange and red, respectively. (C) Gene tracks of H3K4me3 ChIP-seq occupancy. The promoter of *ANKRD30B* and *SLAMF6* genes is the most significantly differentially enriched by H3K4me3 modifications in lenalidomide-sensitive group compared to lenalidomide-resistant group and, conversely, the promoter of *GPR15* and *NKX6-1* genes is the most significantly differentially enriched by H3K4me3 modifications in lenalidomide-resistant group compared to lenalidomide-sensitive group. The x axis shows the genomic position and the y axis shows signal coverage of ChIP-seq occupancy in units of reads per bin mapped reads. (D) Molecular signature of differentially bound sites localized on gene promoters enriched in lenalidomide-sensitive and -resistant HMCLs was investigated using GSEA database (FDR ≤ 0.05). No overlap was found for gene promoters enriched in lenalidomide-sensitive HMCLs.

Of major interest, we identified 5903 significantly differentially H3K4me3-enriched sites between lenalidomide-resistant (AMO1, JJN3, KMS-12-BM, SKMM2, XG1, XG5, XG7, XG12, XG20 and XG21 HMCLs) and lenalidomide-sensitive cell lines (OPM2, RPMI8226, XG2, XG6, XG13 and XG19 HMCLs) (Figure 6A) (FDR ≤ 0.05). Regarding gene promoters, 203 H3K4me3 differentially bound sites were significantly enriched in lenalidomide-sensitive HMCLs compared to lenalidomide-resistant HMCLs (FDR ≤ 0.05) whereas 136 H3K4me3 differentially bound sites were significantly enriched in the lenalidomide-resistant group compared to the lenalidomide-sensitive group (Figure 6B). Examples of significantly differentially enriched regions are presented in Figure 6C. Gene set enrichment analysis revealed that H3K4me3 differentially bound sites enriched in lenalidomide-resistant HMCLs were associated to interferon signaling and cytokine signaling in the immune system (Figure 6D). Differential sites enriched in lenalidomide-sensitive HMCLs were significantly associated with an NFKBIA target gene signature.

**Figure 7:**
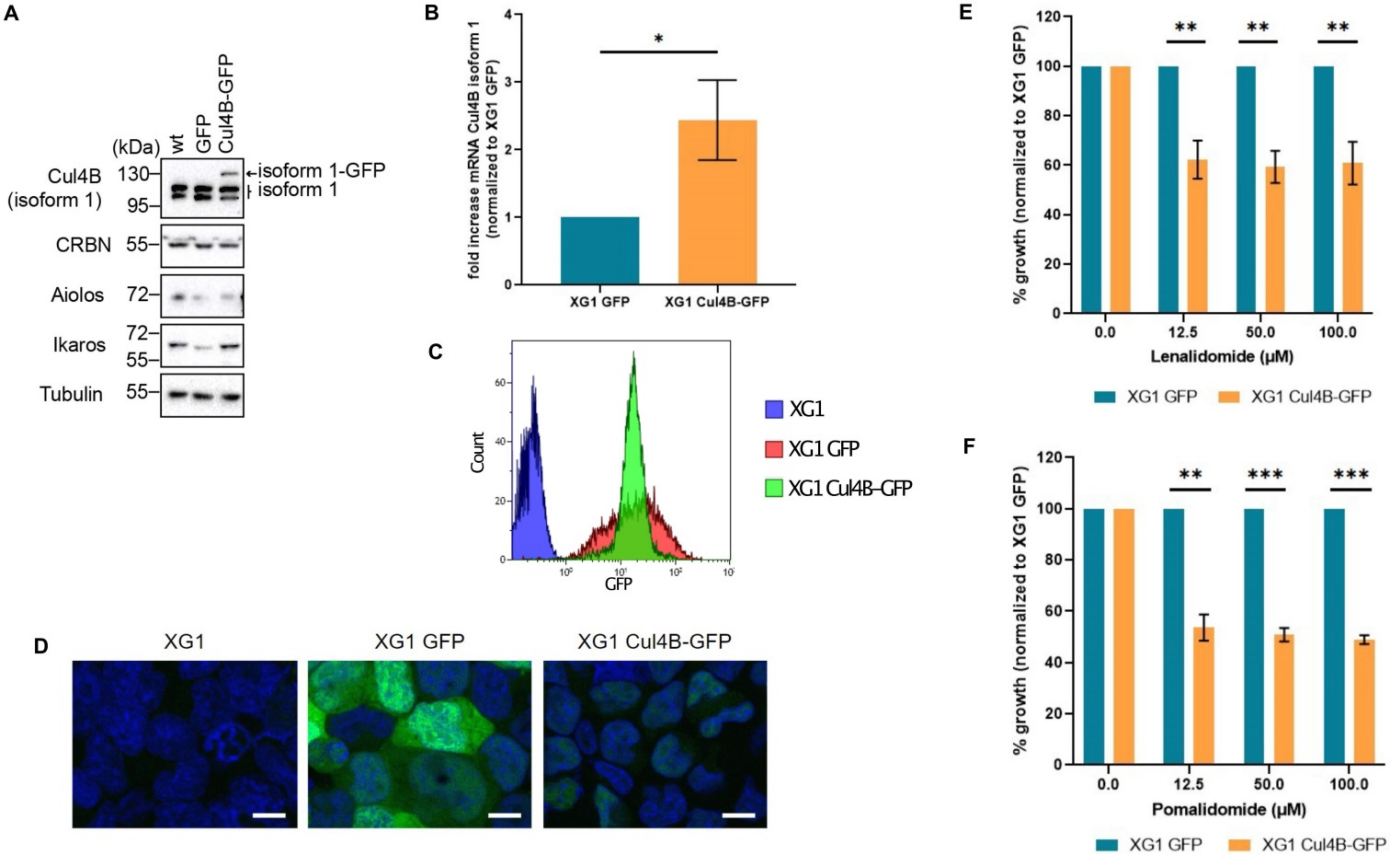
Increased expression of Cul4B isoform 1 re-sensitizes MM cell lines to lenalidomide. XG1 cells were infected with lentiviral particles containing GFP or GFP-tagged Cul4B isoform 1 (Cul4B-GFP), selected with puromycin and sorted by FACS based on GFP expression levels. (A) Protein expression levels of endogenous and ectopic Cul4B isoform 1 analyzed by western blot with a specific antibody in XG1 cells. Protein levels of CRBN were also analyzed. Tubulin used as loading control. (B) The level of Cul4B isoform 1 mRNA in XG1 Cul4B-GFP cells was analyzed by qPCR and normalized to the level in XG1 GFP cells. N = 3. t-test p-value = 0.05. (C) GFP and Cul4B-GFP expression levels in XG1 cells were analyzed by flow cytometry. (D) Cells were fixed with 4% PFA for 10 min at RT. DNA was stained with DAPI (20μg/ml) for 5 min. Scale bar = 10 um. (E) Cells were treated with the indicated concentrations of lenalidomide for 4 days. Cell growth was analyzed by Cell Titer Glo (CTG) and normalized to XG1 GFP for each concentration. Graph shows the average of 4 independent experiments. T-test significance: p-value (12.5 μM) = 0.0023, p-value (50 μM) = 0.0011, p-value (100 μM) = 0.0028. (F) Cells were treated with the indicated concentrations of pomalidomide for 4 days and proliferation was analyzed by Cell Titer Glo (CTG). Graph shows the average of 3 independent experiments. p-value (12.5 μM) = 0.004, p-value (50 μM) = 0.0009, p-value (100 μM) = 0.0004.

Lenalidomide targets the Cereblon complex, formed by CUL4, RBX1, DDB1 and CRBN proteins 59. Interestingly, we observed a H3K4me3 enrichment on the promoter of *CUL4B* in lenalidomide-sensitive compared to resistant HMCLs (Supplementary Figure 15). The presence of *CUL4B* isoform 1 appeared to be associated with lenalidomide sensitivity. To validate this observation, we generated XG1 cell line derivatives stably expressing GFP tagged-Cul4B isoform 1 (Cul4B-GFP cell line) or the GFP alone. The overexpression of Cul4B isoform1 expression was validated both by western blot and RT-qPCR (Figure 7A and 7B). Cells were sorted by flow cytometry based on their GFP levels in order to keep those with high expression of Cul4B-GFP (Figure 7C). Nuclear localization of the ectopic Cul4B-GFP was confirmed by microscopy (Figure 7D). Sensitivity to lenalidomide of the XG1 Cul4B-GFP and the control XG1-GFP cell lines were studied by proliferation assays. MM cells expressing Cul4B-GFP were significantly more sensitive to lenalidomide compared to the control (Figure 7E). Moreover, Cul4B-GFP expression also increased cell sensitivity to pomalidome, a third generation IMiD that also targets CRBN 59 (Figure 7F). Altogether, these data indicate that the expression of Cul4B isoform 1 is related to the response to IMiDs in MM cells and support the potential interest of ChIP-seq analyses to identify biomarkers that could predict response to these drugs.

To confirm the potential of ChIP sequencing data to identify epigenetic mechanisms involved in drug resistance, we investigate the bound sites differentially enriched in H3K4me3 between romidepsin-resistant (AMO1, XG7, XG13, XG19 and XG20) and romidepsin-sensitive (OPM2, RPMI8226, SKMM2, XG1, XG5, XG6 and XG12) cell lines (Supplementary Figure 16A) (FDR ≤ 0.05). Among the 2098 H3K4me3 significantly differentially bound sites, 250 bound sites were localized on gene promoters (Supplementary Figure 16B). Romidepsin is a class I histone deacetylase inhibitor currently evaluated in combination with lenalidomide in phase I/II (NCT017755975) in patients with relapse or refractory multiple myeloma. Among the 250 H3K4me3 bound sites located on gene promoters, 180 were significantly enriched in romidepsin-sensitive HMCLs associated with an HDAC3 and PRDM6 target gene signature (Supplementary Figure 16D). We found 70 differentially bound sites enriched in the romidepsin-resistant group compared to the romidepsin-sensitive group. Gene set enrichment analysis revealed a significant association with H3K27me3 and polycomb complex subunit (SUZ12 and EED) target genes. That indicates that ChIP-seq strategies could be an interesting tool to identify biomarkers associated with drug response or resistance in MM. Further investigations are needed to establish potential future clinical applications.

Moreover, among the 6 histones modifications, only the differentially bound sites identified with H3K4me1, H3K4me3 and H3K9me3 allowed the distinction between resistant and sensitive cell lines for lenalidomide and romidepsin drugs (Supplementary Figure 17 and 18). Using H3K9me3, only few bound sites were significantly differentially enriched between resistant and sensitive groups. It could be interesting to carry out more investigations on the H3K4me1 differentially bound sites to identify super-enhancers associated to drug resistance. Altogether, these data underline the interest of ChIP sequencing to identify epigenetic biomarkers related to drug response in MM.

## Discussion

In this study, we have characterized the landscape of histone modifications in a large collection of HMCLs representative of MM molecular heterogeneity 16. Some histone modification profiles, such as H3K27me3, were clearly related to cytogenetic abnormalities. In t(4;14) HMCLs, *MMSET* overexpression results in the genome-wide redistribution of the SET domain protein EZH2 and its associated H3K27me3 mark 60. Gene expression profiling revealed deregulated genes involved in cell cycle (e.g. *CCNE2*), apoptosis (e.g. *BAX* and *BCL2*) and DNA repair (e.g. *ATM* and *GADD45A*) 61. Moreover, we showed that promoter of genes involved in the negative regulation of cell cycle (e.g. *CEND1* and *CDKN1C*) and the induction of apoptosis (e.g. *TP73* and *BNIP3*) were enriched in the H3K27me3 mark in t(4;14) HMCLs. Methylation of the *BNIP3* promoter was also described and associated with poor overall survival in MM patients 62. These results confirmed the interplay between MMSET, EZH2 and H3K27me3 61,63 and revealed new potential therapeutic targets for t(4;14) MM patients associated with poor prognosis 64. We also observed that *TP53* bi-allelic events are associated with a H3K9me3 signature. SUV39H1, the histone methyltransferase responsible for the H3K9me3 mark, is a target of p53 repression and p53 target promoters are enriched in the H3K9me3 repressive mark 65, suggesting that *TP53* mutation could modulate H3K9me3 level on these promoters and thus the p53 apoptotic response.

We and others showed that super-enhancers were associated to key genes contributing to myeloma pathogenesis, such as *MYC*, *IRF4*, *CCND1*, *NSD2* and *MAF* 39,66. Acetylated chromatin, particularly super-enhancer regions, is associated to bromodomain and extra-terminal (BET) proteins to facilitate transcriptional activation 67. BET inhibitors disrupt the interaction of bromodomains with acetylated histones and lead to loss of enhancer-promoter long-range interactions. The score based on genes associated with super-enhancers built in this study allows one to identify high-risk MM patients that might benefit from BET inhibitors. Several BET inhibitors are currently evaluated in phase I/II in MM patients, including OTX015 (NCT01713582), CPI-0610 (NCT02157636), GSK525762 (NCT01943851), and RO6870810 (NCT03068351) 68. We showed that SE-associated risk score was correlated with the response to GSK525762 in HMCLs. Moreover, concerning the 28 genes composing the SE-risk score, the prognostic value of 7 genes (*BSG*, *HK2*, *HSPA9*, *IL10*, *PARP1*, *PDIA6* and *SLC7A5*) has already been described in MM 69-79 while the expression of *HNRNPC*, *ILF3*, *LDHB*, *MDH1*, *NCL*, *PRPS1*, *RPL8*, *RPL13A*, *RPL35*, *SMARCA4*, *SPN, STC2 and THY1* was described to be associated to tumorigenesis, drug resistance and/or poor prognosis in other cancers 80-93. Furthermore, the prognostic value of *MYBPC2*, *NUDC*, *RPL27A*, *SF3BS*, *SLC25A39*, *TNPO2*, *TPR*, *YWHAQ* transcriptional deregulation has not been described before. Interestingly, among these genes associated with SE in MM, *YWHAQ*, *IL10*, *HK2* and *THY1* were identified as significant essential MM genes in RNAi or CRISPR screening, corroborating epigenetic and transcriptional modifications at a functional level.

It is widely believed that H3K9me3 and H3K27me3 do not co-occur at the same loci, but some ChIP-seq data indicate that these two marks can be found together at a subset of developmentally regulated gene in mouse embryonic stem cells, extra-embryonic lineages and human differentiated cells 54-56. This dual repression at specialized regulators may point to the importance of maintaining their silencing to confer a selective advantage in tumoral cells. Here, we identified 18 potential suppressor tumor genes enriched in H3K9me3 and H3K27me3 repressive marks on their promoter. To our knowledge, the tumor suppressor function of these genes has not yet been described in MM. However, some of them are involved in cell proliferation inhibition. The *NLRP2* gene, coding for a member of the nucleotide binding and leucine-rich repeat receptor (NLR), is an inhibitor of NF-κB pathway inhibitor which plays a key role in survival and proliferation of myeloma cells 94,95. *PAG1* encodes for a type III transmembrane adaptor protein and is an inhibitor of Src tyrosine kinases known to promote proliferation in MM 96,97. From these 18 genes we built a score identifying a high-risk group of MM patients with global low expression of putative suppressor genes associated with H3K9me3 and H3K27me3 marks. We showed that this score was correlated with the response to EPZ-3864 and chaetocin, a SUV39H1/2 protein inhibitor, in HMCLs. High-risk MM patients identified with this score could benefit from combined therapeutic targeting of EZH2 and SUV39H1 histone methyltransferases. Moreover, the therapeutic interest of EZH2 inhibitors has already been demonstrated in HMCLs and primary myeloma samples 98. Concerning SUV39H1, high expression level is associated with a poor prognosis in MM patients and SUV39H1 inhibitor also exhibited anti-MM effects both in HMCLs and primary samples 99.

Despite the improvement of MM patient survival through the development of novel agents, including new generation of IMiDs or proteasome inhibitors, monoclonal antibodies and HDAC inhibitors, the acquisition of drug resistance is the major limitation of MM therapy. The great majority of MM patients ultimately relapse and the treatment of relapse/refractory MM remains a major challenge. In this study, we analyzed the relationship between differential enrichment of H3K4me3 on gene promoters in HMCLs and response to treatment. We developed an epigenetic biomarker based on this histone modification, which predicts lenalidomide and romidepsin responses in HMCLs. IMiDs are known to target the cereblon complex. Cereblon is composed of CUL4, RBX1, DDB1 and CRBN proteins and induces the ubiquitination of B-cell transcription factors IKZF1 and IKZF3 in presence of IMiDs 59. Among the genes encoding these proteins, we observed a significant difference in H3K4me3 enrichment on the promoter of *CUL4B* in lenalidomide-sensitive HMCLs compared to resistant HMCLs (Supplementary Figure 15). In other studies, loss of *CUL4B* has been described as conferring resistance to lenalidomide in lymphoma and myeloma cell lines 100,101. Here, we have shown that the presence of the *CUL4B* splicing variant 1 is indeed associated with sensitivity to lenalidomide and pomalidomide.

This study provides a comprehensive characterization of the MM epigenetic landscape, representing a unique resource for future biological studies which could help in identifying novel critical epigenetic modifications involved in MM progression and drug resistance. Furthermore, risk-scores based on super enhancers and repressive regions together with epigenetic biomarkers of drug response could represent new tools for precision oncology in MM.

## Supplementary Material

Supplementary figures and tables.

## Figures and Tables

**Table 1:** Characteristics of HMCLs based on molecular classification

HMCLs name	HMCL classification	Target genes	Translocation	del1p	1qgain	del13q	del17p
AMO-1	CD-2L	*CCND2*	t(12;14)	+	+	-	-
JJN3	MF	*c-Maf*	t(14;16)	-	+	+	+
KMS-12-BM	CD-2L	*CCND1*	t(11;14)	+	-	+	+
OPM2	MS	*MMSET*/*FGFR3*	t(4;14)	+	+	-	+
RPMI8226	MF	*c-Maf*	t(14;16)	+	+	+	+
SKMM2	CD-1	*CCND1*	t(11;14)	+	-	+	+
XG1	CTA/FRZB	*CCND1*	t(11;14)	-	-	-	-
XG2	CTA/FRZB	*CCND2*	t(12;14)	-	-	-	-
XG5	CD-1	*CCND1*	t(11;14)	+	-	-	+
XG6	CTA/MF	*c-Maf*	t(16;22)	+	-	+	-
XG7	MS	*MMSET*	t(4;14)	+	+	+	-
XG12	CTA/MF	*c-Maf*	t(14;16)	+	-	-	-
XG13	MF	*c-Maf*	t(14;16)	+	-	+	+
XG19	CTA/MF	*c-Maf*	t(14;16)	+	-	+	+
XG20	MS	*MMSET*	t(4;14)	+	-	-	+
XG21	CD-1	*CCND1*	t(11;14)	-	+	-	-
