# Supplementary material for: Comprehensive characterization of the epigenetic landscape in Multiple Myeloma
Source: Theranostics. 2022 Jan 16;12(4):1715–29. doi: 10.7150/thno.54453 (PMC8825580; doi:10.7150/thno.54453)
Supplement: Supplementary file 1 — Supplementary figures and tables. [file thnov12p1715s1.pdf]

Supplementary Table 1: Comparison of the peaks of the six histones modifications in 4 MM patients and 16 HMCLs. Liftover function was used to change the genomic coordinate of peaks identified in MMC of MM patients. Overlapping regions of MMC peaks and HMCLs peaks were identified using the findOverlaps function.

	H3K4me1	H3K4me3	H3K9me3	H3K27ac	H3K27me3	H3K36me3
Percentage of MMC peaks found in HMCL peaks	98.05%	83.93%	82.16%	69.11%	89.02%	85.69%

Supplemental Table 2: Correlation values of the differential H3K9me3 enrichment analysis in HMCLs using Diffbind R package. The correlations range from 0 (no correlation) to 1 (strong correlation). Red boxes correspond to HMCLs composing the main cluster identified by the unsupervised hierarchical clustering.

	XG5	KMS12BM	OPM2	RPMI	XG19	SKMM2	XG1	XG21	XG2	XG6	XG13	JJN3	XG12	XG20	XG7	AMO1
XG5	1	0.21	0.15	0.19	0.091	0.14	0.52	0.48	0.4	0.42	0.48	0.27	0.35	0.31	0.38	0.35
KMS12BM	0.21	1	0.56	0.55	0.53	0.55	0.37	0.37	0.42	0.43	0.45	0.48	0.48	0.41	0.37	0.44
OPM2	0.15	0.56	1	0.61	0.61	0.65	0.36	0.38	0.46	0.5	0.5	0.53	0.56	0.52	0.47	0.51
RPMI	0.19	0.55	0.61	1	0.64	0.63	0.4	0.47	0.53	0.47	0.53	0.56	0.61	0.56	0.53	0.62
XG19	0.091	0.53	0.61	0.64	1	0.67	0.31	0.41	0.49	0.4	0.44	0.5	0.54	0.51	0.45	0.51
SKMM2	0.14	0.55	0.65	0.63	0.67	1	0.33	0.44	0.51	0.45	0.47	0.56	0.55	0.55	0.46	0.52
XG1	0.52	0.37	0.36	0.4	0.31	0.33	1	0.53	0.49	0.52	0.57	0.47	0.54	0.41	0.47	0.49
XG21	0.48	0.37	0.38	0.47	0.41	0.44	0.53	1	0.6	0.5	0.56	0.43	0.54	0.51	0.56	0.61
XG2	0.4	0.42	0.46	0.53	0.49	0.51	0.49	0.6	1	0.55	0.58	0.49	0.56	0.56	0.59	0.61
XG6	0.42	0.43	0.5	0.47	0.4	0.45	0.52	0.5	0.55	1	0.66	0.49	0.55	0.5	0.53	0.54
XG13	0.48	0.45	0.5	0.53	0.44	0.47	0.57	0.56	0.58	0.66	1	0.53	0.63	0.54	0.55	0.6
JJN3	0.27	0.48	0.53	0.56	0.5	0.56	0.47	0.43	0.49	0.49	0.53	1	0.58	0.53	0.51	0.56
XG12	0.35	0.48	0.56	0.61	0.54	0.55	0.54	0.54	0.56	0.55	0.63	0.58	1	0.55	0.55	0.63
XG20	0.31	0.41	0.52	0.56	0.51	0.55	0.41	0.51	0.56	0.5	0.54	0.53	0.55	1	0.64	0.65
XG7	0.38	0.37	0.47	0.53	0.45	0.46	0.47	0.56	0.59	0.53	0.55	0.51	0.55	0.64	1	0.71
AMO1	0.35	0.44	0.51	0.62	0.51	0.52	0.49	0.61	0.61	0.54	0.6	0.56	0.63	0.65	0.71	1

Supplemental Table 3: Correlation values of the differential H3K27me3 enrichment analysis in HMCLs using Diffbind R package. The correlations range from 0 (no correlation) to 1 (strong correlation). Red boxes correspond to HMCLs composing the main cluster identified by the unsupervised hierarchical clustering.

	XG7	XG20	OPM2	AMO1	RPMI	KMS12BM	XG19	XG2	XG1	JJN3	SKMM2	XG21	XG5	XG12	XG6	XG13
XG7	1	0.51	0.5	0.26	0.29	0.26	0.35	0.37	0.3	0.33	0.34	0.31	0.34	0.37	0.38	0.43
XG20	0.51	1	0.6	0.3	0.33	0.33	0.44	0.47	0.38	0.44	0.45	0.37	0.42	0.43	0.43	0.54
OPM2	0.5	0.6	1	0.29	0.35	0.35	0.45	0.5	0.37	0.48	0.46	0.37	0.43	0.46	0.45	0.54
AMO1	0.26	0.3	0.29	1	0.4	0.43	0.34	0.42	0.42	0.4	0.4	0.49	0.42	0.49	0.44	0.48
RPMI	0.29	0.33	0.35	0.4	1	0.43	0.44	0.42	0.37	0.43	0.44	0.4	0.37	0.43	0.42	0.49
KMS12BM	0.26	0.33	0.35	0.43	0.43	1	0.43	0.46	0.41	0.4	0.43	0.44	0.43	0.46	0.45	0.5
XG19	0.35	0.44	0.45	0.34	0.44	0.43	1	0.53	0.45	0.48	0.52	0.44	0.45	0.49	0.44	0.57
XG2	0.37	0.47	0.5	0.42	0.42	0.46	0.53	1	0.47	0.51	0.51	0.48	0.49	0.51	0.53	0.61
XG1	0.3	0.38	0.37	0.42	0.37	0.41	0.45	0.47	1	0.51	0.53	0.49	0.52	0.5	0.44	0.57
JJN3	0.33	0.44	0.48	0.4	0.43	0.4	0.48	0.51	0.51	1	0.55	0.44	0.47	0.45	0.43	0.59
SKMM2	0.34	0.45	0.46	0.4	0.44	0.43	0.52	0.51	0.53	0.55	1	0.54	0.54	0.46	0.45	0.61
XG21	0.31	0.37	0.37	0.49	0.4	0.44	0.44	0.48	0.49	0.44	0.54	1	0.53	0.49	0.45	0.55
XG5	0.34	0.42	0.43	0.42	0.37	0.43	0.45	0.49	0.52	0.47	0.54	0.53	1	0.46	0.46	0.6
XG12	0.37	0.43	0.46	0.49	0.43	0.46	0.49	0.51	0.5	0.45	0.46	0.49	0.46	1	0.53	0.59
XG6	0.38	0.43	0.45	0.44	0.42	0.45	0.44	0.53	0.44	0.43	0.45	0.45	0.46	0.53	1	0.66
XG13	0.43	0.54	0.54	0.48	0.49	0.5	0.57	0.61	0.57	0.59	0.61	0.55	0.6	0.59	0.66	1

Supplementary Table 4: List of genes deregulated in t(4;14) HMCLs compared to HMCLs without t(4;14) translocation, with the promoter differentially enriched in H3K27me3

**Genes overexpressed with the promoter enriched in H3K27me3**		
ACTN1	*HS3ST3B1*	*OTULINL*
ADD2	*IL10RA*	*PIMREG*
ARHGEF11	*KCTD1*	*RBFOX2*
BAIAP2L1	*KCTD12*	*RUNX1*
DAB2IP	*LRP6*	*SPRED2*
DUSP23	*MAF*	*SULF1*
ENAH	*MAGI1*	*TENM3*
ETV5	*MAP10*	*TTC7B*
EVL	*MBP*	*ZNF516*
GALNT11	*MYH10*	
GTF2IRD1	*NCAM1*	

**Genes downregulated with a lower H3K27me3 level on promoter**
DPP7
ICAM1
ZNF615

Supplemental Table 5: Super-enhancers and SE-associated genes detected in HMCLs

HMCLs name	Activated SE	SE-associated genes (<50kb)	SE-associated genes with strong expression
AMO1	994	2493	96
JJN3	1485	3202	95
KMS-12-BM	1770	5300	97
OPM2	2211	5300	163
RPMI8226	1187	3191	106
SKMM2	1293	3926	88
XG1	1959	4584	121
XG12	2510	5425	145
XG13	2030	4815	108
XG19	2293	5764	145
XG2	1368	3164	86
XG20	1782	3727	119
XG21	607	1540	67
XG5	631	1132	34
XG6	1643	3656	112
XG7	2352	5299	153

Supplemental Table 6: DEMETER2 and CERES score comparison between MM cell lines and other cancer cell lines. DEMETER2 and CERES scores were calculated from CRISPR-Cas9 and RNAi-based viability assays, respectively.

Gene	CRISPR		RNAi	
	T-Statistic	P-Value	T-Statistic	P-Value
YWHAQ	-	-	-5.2950724	1.71E-07
THY1	-	-	-5.5790531	3.78E-08
IL10	-4.4392273	1.04E-05	-	-
HK2	-4.0703292	5.19E-05	-	-

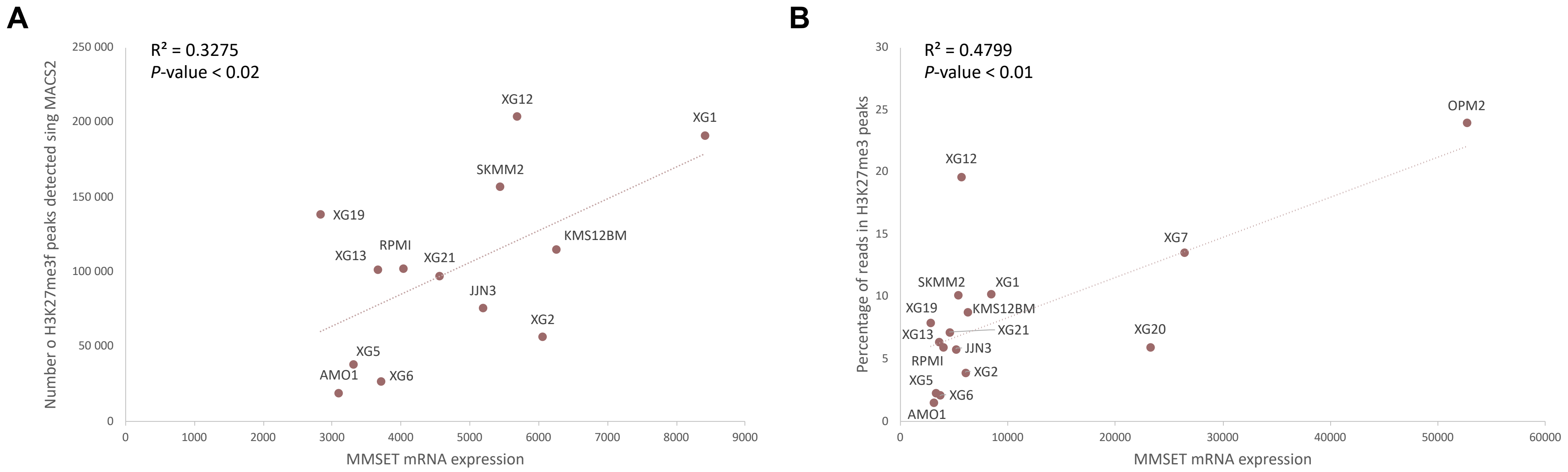

Supp Figure 1. Correlation between MMSET mRNA expression and H3K27me3 histone modification in HMCLs. (A) Linear regression analysis of the number of H3K27me3 peaks in function of the the MMSET mRNA expression in 13 HMCLs without t(4;14) translocation. (B) Linear regression analysis of the percentage of reads in H3K27me3 peaks (FRiP) in function of the MMSET mRNA expression in 16 HMCLs (13 HMCLs without t(4;14) translocation and 3 HMCLs harboring t(4;14) translocation). Coefficient of determination R^2 represents the square of the Pearson correlation coefficient (r) (Pearson correlation test).

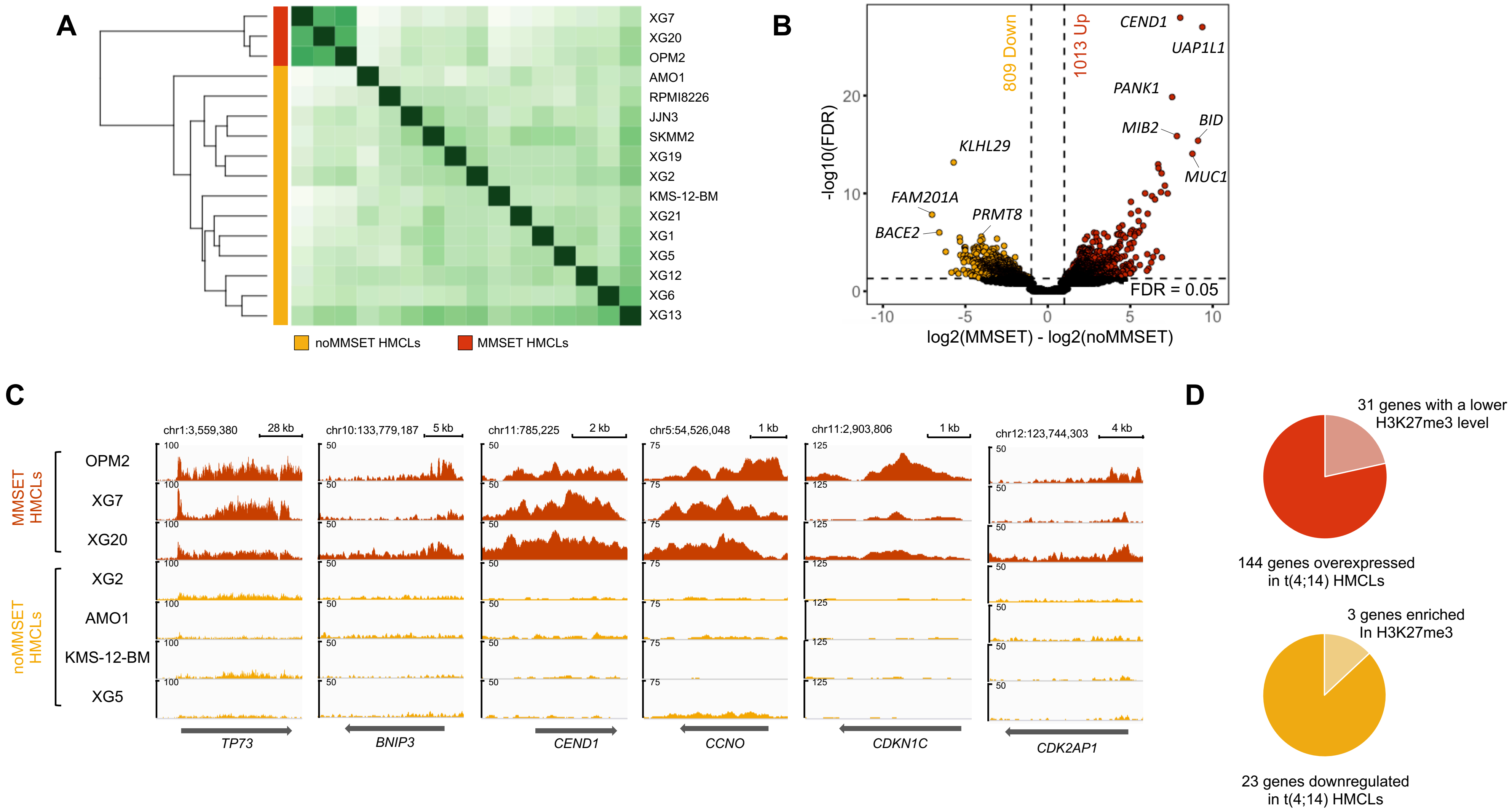

Supp Figure 2.

Supp Figure 2. H3K27me3 modification differentially enriched in HMCLs with and without t(4:14) translocation. (A) Correlation heatmap using only the 45992 sites identified as being significantly differentially bound in MMSET (with t(4;14) translocation) compared to noMMSET (without t(4;14) translocation) HMCLs (FDR ≤ 0.05). (B) Volcano plot of differentially bound sites localized on promoters using MMSET *vs* noMMSET HMCLs contrast. Sites identified as significantly differentially bound in noMMSET and MMSET HMCLs are colored in orange and red, respectively. (C) Gene tracks of H3K27me3 ChIP-seq occupancy. *TP73* and *BNIP3* played a role in apoptosis and *CEND1*, *CCNO*, *CDKN1C* and *CDK2AP1* were involved in the negative regulation of cell cycle. The x axis shows the genomic position and the y axis shows signal coverage of ChIP-seq occupancy in units of reads per bin mapped reads. (D) Genes deregulated in t(4;14) HMCLs compared to HMCLs without t(4;14) translocation and the proportion of genes with the promoter differentially enriched in H3K27me3.

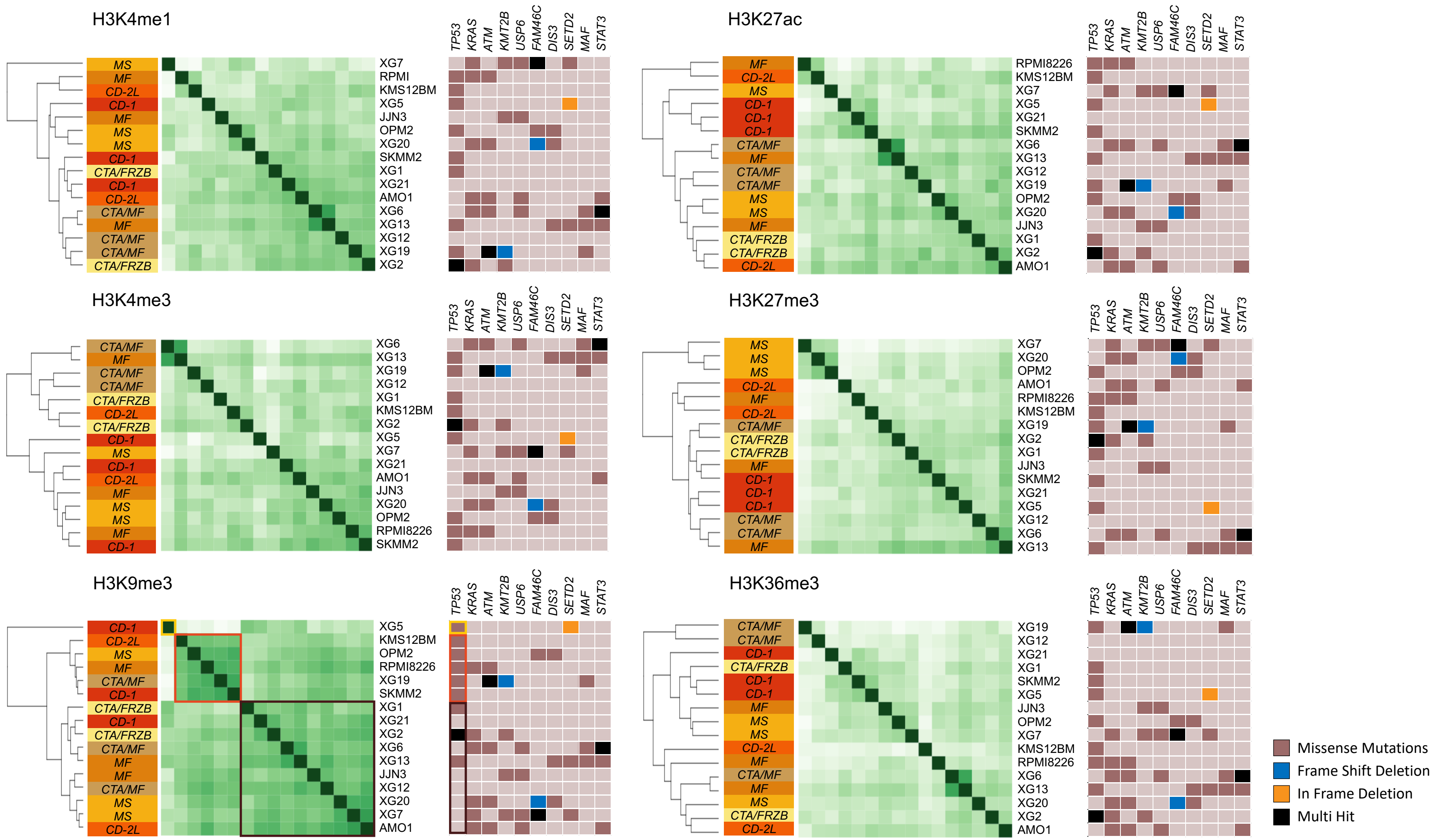

Supp Figure 3.

Supp Figure 3. Link between recurrent mutations and H3K4me1, H3K4me3, H3K9me3, H3K27ac, H3K27me3 and H3K36me3 ChIP-seq profiles in 16 HMCLs. Correlation heatmaps performed using affinity data (read count) suggest a link between H3K9me3 ChIP-seq profile and *TP53* mutation. Yellow, orange and brown boxes encompass interest clusters.

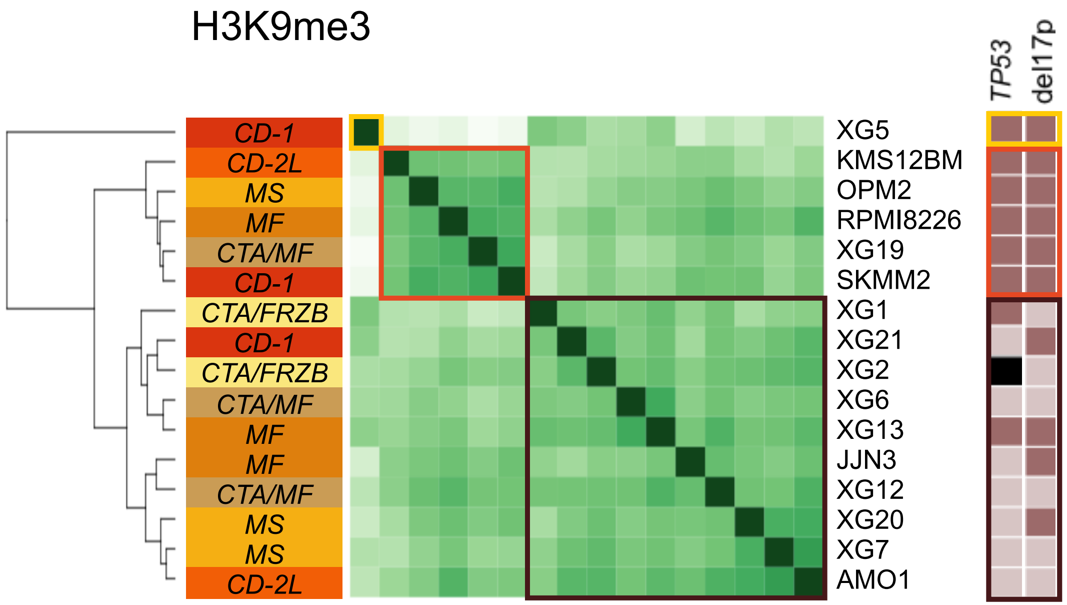

Supp Figure 4. H3K9me3 correlation heatmaps in 16 HMCLs reveal a link between H3K9me3 ChIP-seq profile and TP53^{mut} and del17p double hit. Yellow, orange and brown boxes encompass interest clusters. Dark and light boxes correspond to the presence or the absence of TP53 mutation or del17p cytogenetic abnormality, respectively.

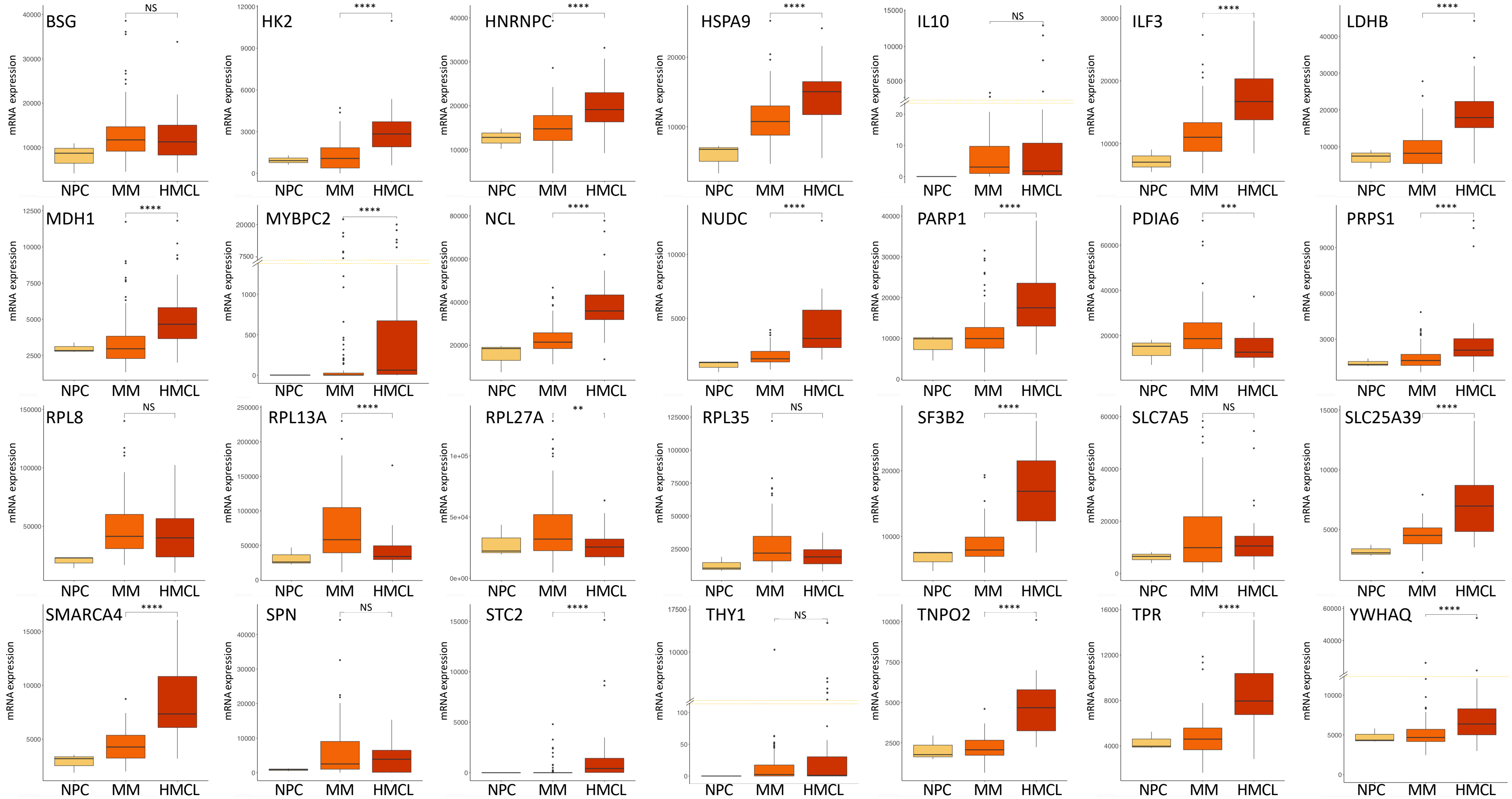

Supp Figure 5.

Supp Figure 5. Boxplots representing mRNA expression of genes composing SE-risk score in normal plasma cells (NPC; n = 3), plasma cells of MM patients (n = 97) and HMCLs (n = 33). Wilcoxon test. NS: non significant, **P*-value < 0.05, ***P*-value < 0.01, ****P*-value < 0.001, *****P*-value < 0.0001.

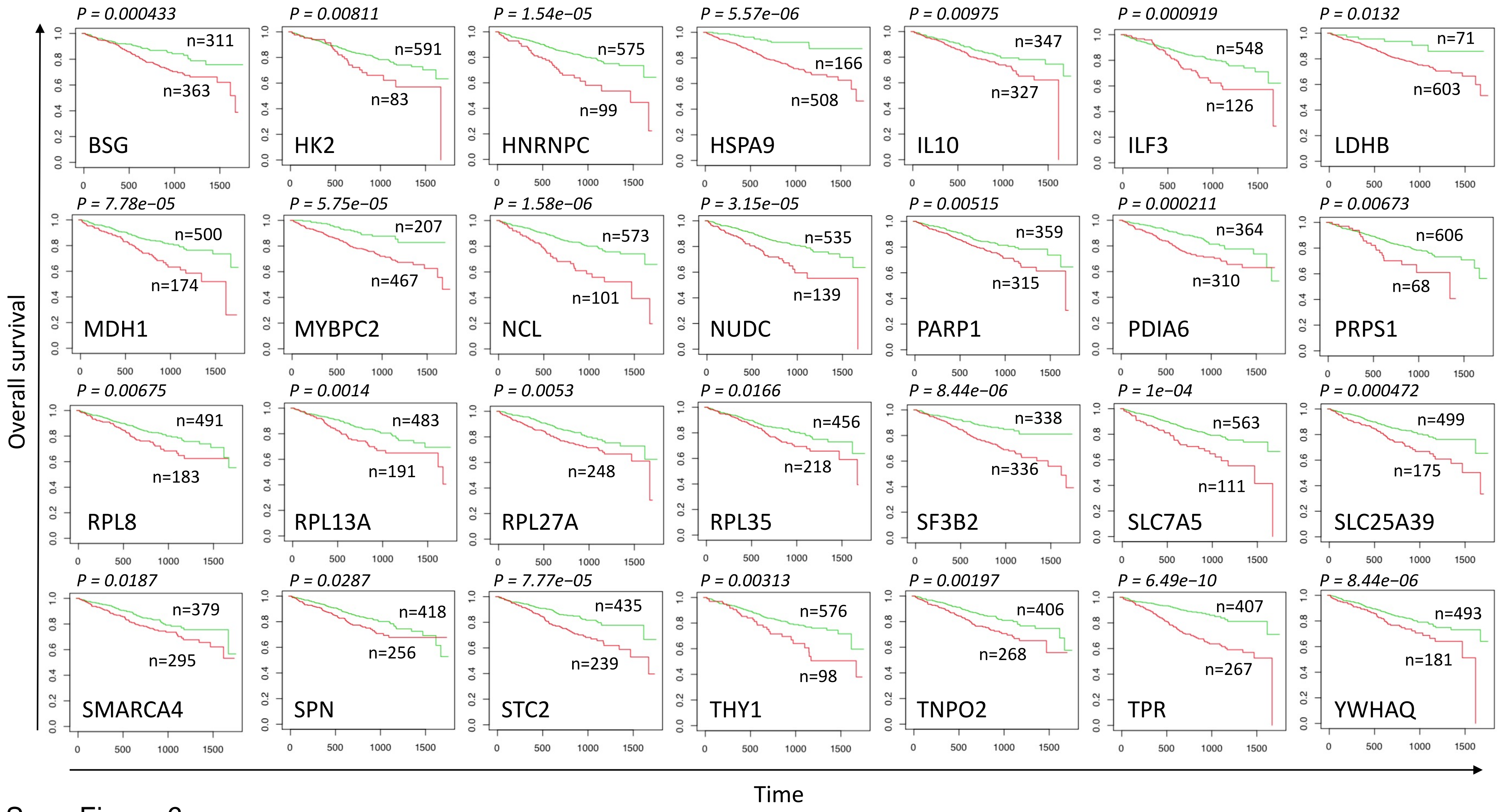

Supp Figure 6.

Supp Figure 6. Kaplan-Meier curves of genes composing SE-risk score in CoMMpass cohort (n = 674). Red curves represent high-risk group associated with high gene expression and green curves correspond to low-risk group associated with low gene expression

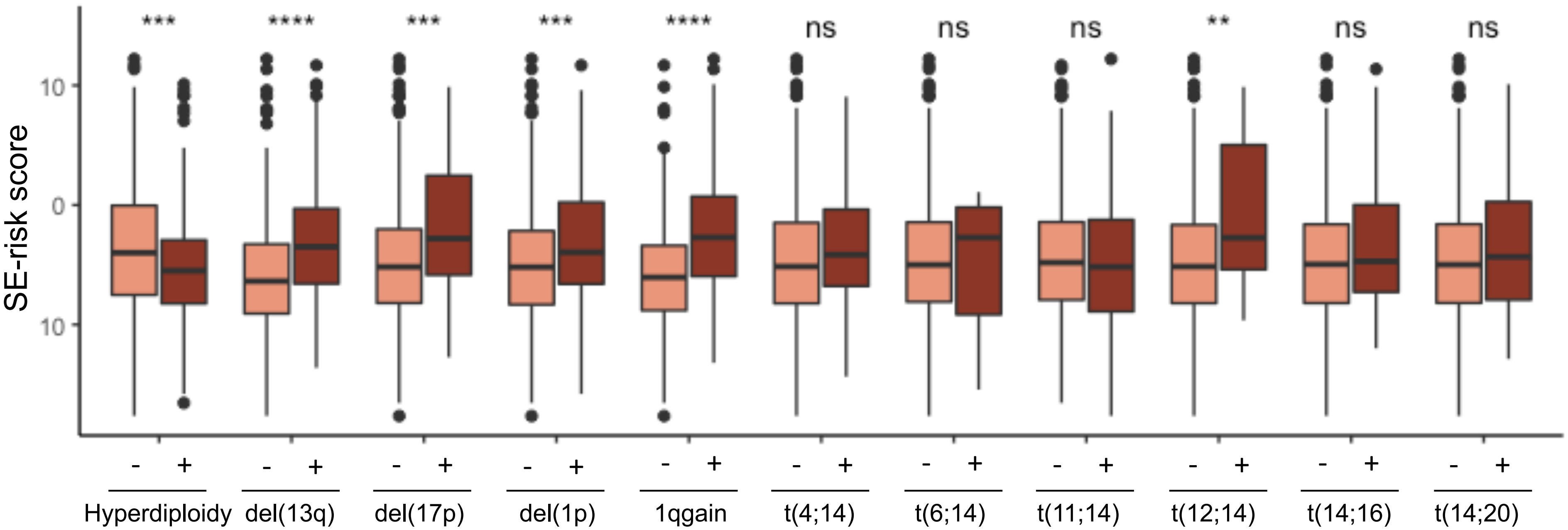

Supp Figure 7. SE-risk score in cytogenetic abnormality subgroups of the CoMMpass cohort. Cytogenetic abnormality subgroups were composed of hyperdyploid (without: n=240, with: n=331), del(13p) (without: n=307, with: n=266), del(17p) (without: n=523, with: n=50), del(1p) (without: n=455, with: n=118), 1q gain (without: n=378, with: n=195) groups of patients and patients harboring t(4;14) (without: n=515, with: n=73), t(6;14) (without: n=572, with: n=16), t(11;14) (without: n=461, with: n=127), t(12;14) (without: n=564, with: n=24), t(14;16) (without: n=532, with: n=56), t(14;20) (without: n=543, with: n=45) translocations. Wilcoxon test. ns: non significant, *P-value < 0.05, **P-value < 0.01, ***P-value < 0.001, ****P-value < 0.0001.

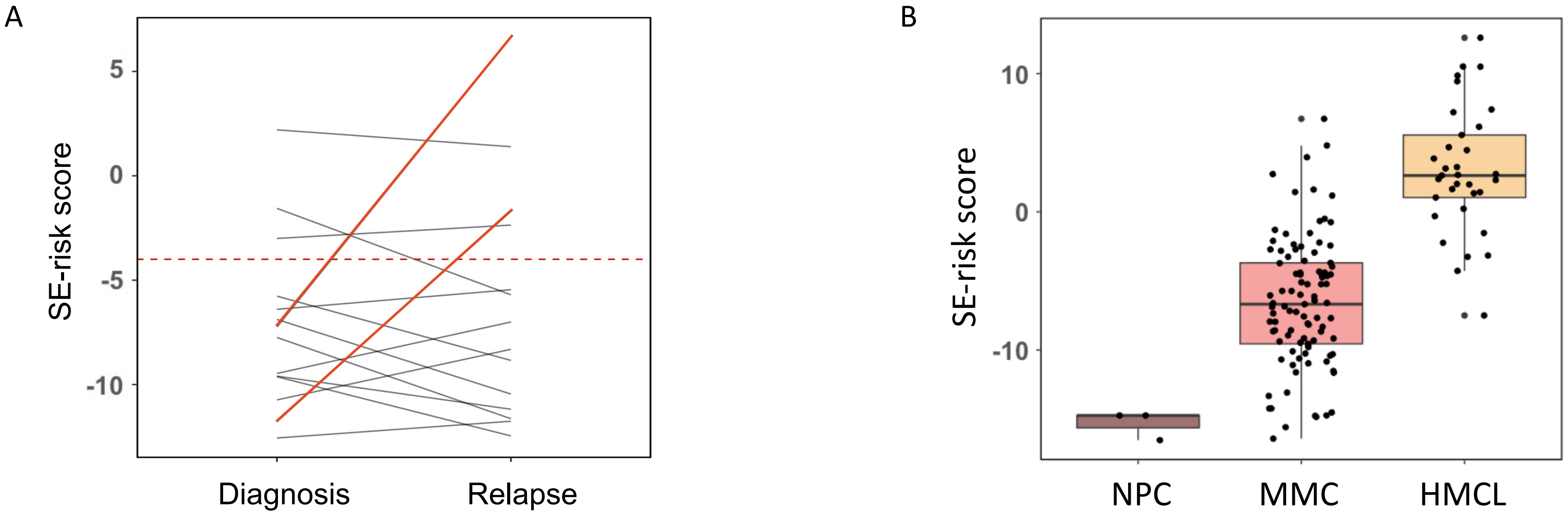

Supp Figure 8. SE-risk score in normal and tumoral plasma cells. (B) Score evolution in 14 paired diagnosis-relapse samples of MM patients from the Montpellier cohort. Solid red curves correspond to MM patients with strong increase in the score. Dashed line corresponds to the cutpoint (-4.0) splitting patients into high-risk and low-risk groups. (A) The score is calculated in normal plasma cells (NPC; n = 3), plasma cells of MM patients (n = 97) and HMCLs (n = 33).

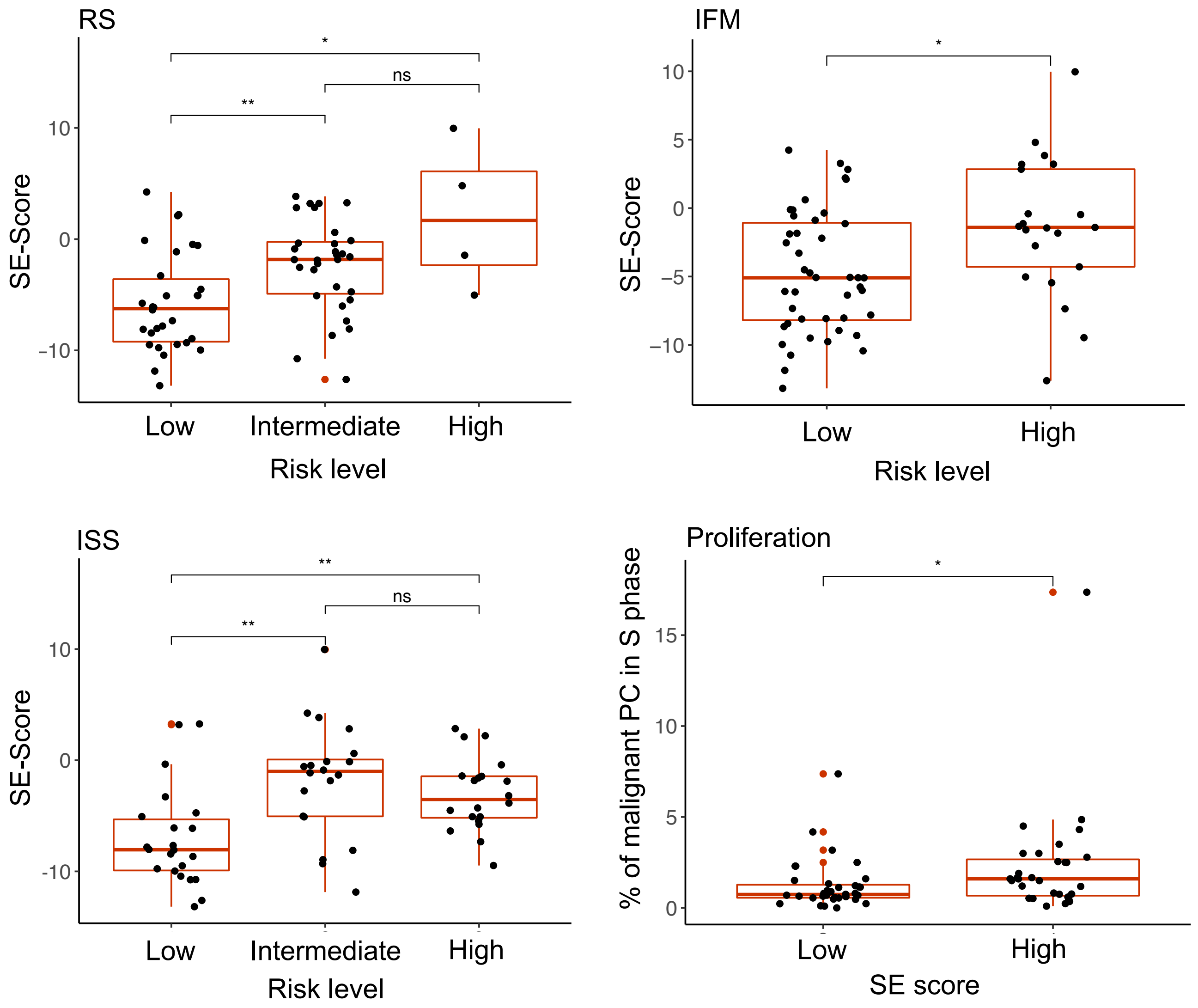

Supp Figure 9. Comparison of SE-risk score with Affymetrix GEP-based risk scores (RS and IFM), ISS and proliferation. Wilcoxon test. ns: non significant, *P-value < 0.05, **P-value < 0.01, ***P-value < 0.001, ****P-value < 0.0001.

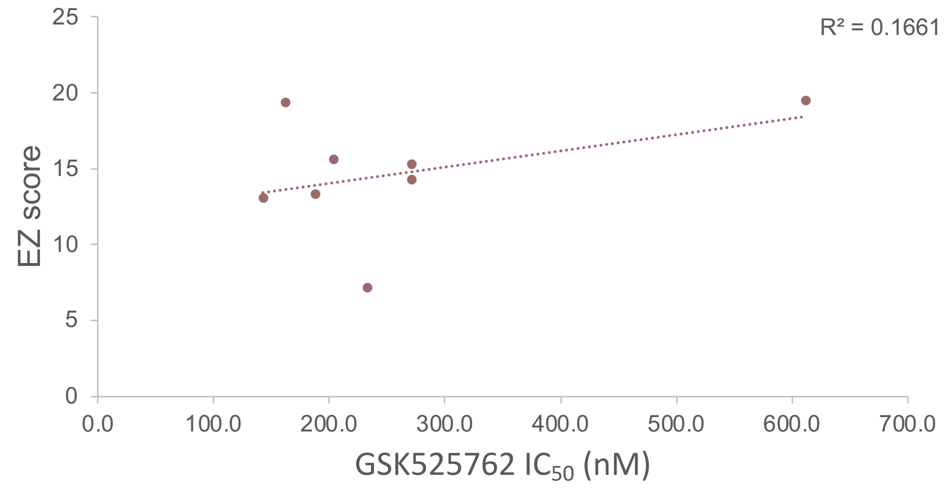

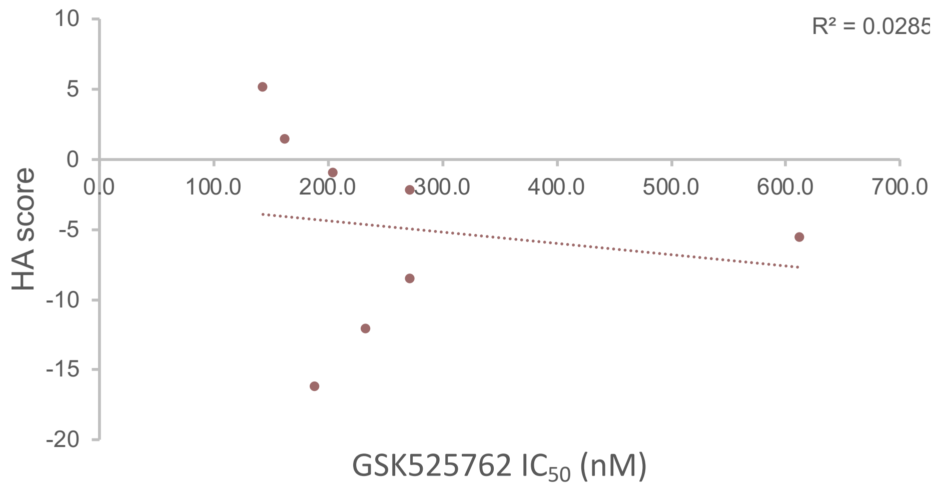

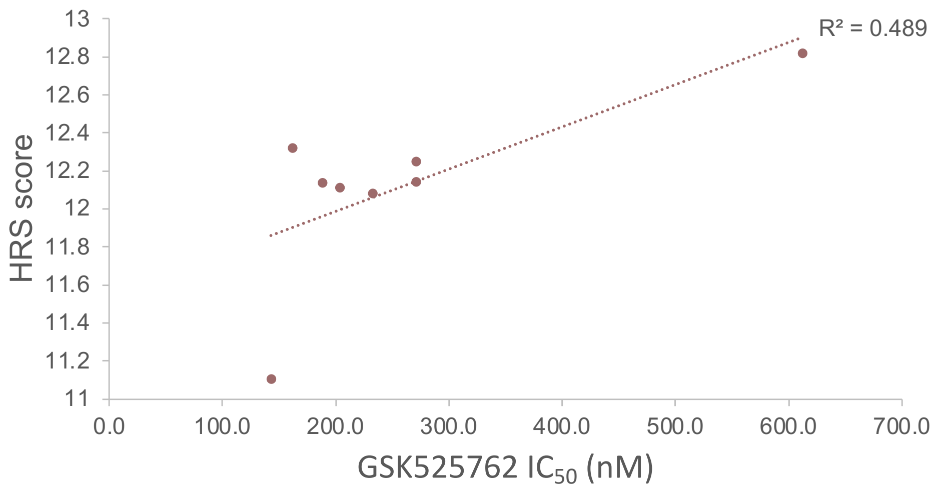

Supp Figure 10. Correlation between epigenetic (EZ and HA) and GEP (HRS) scores calculated in HMCLs and the response to GSK525762. Linear regression analysis of the scores in function of the IC50 of GSK525762 in 8 HMCLs. Coefficient of determination R^2 represents the square of the Pearson correlation coefficient (r) (Pearson correlation test).

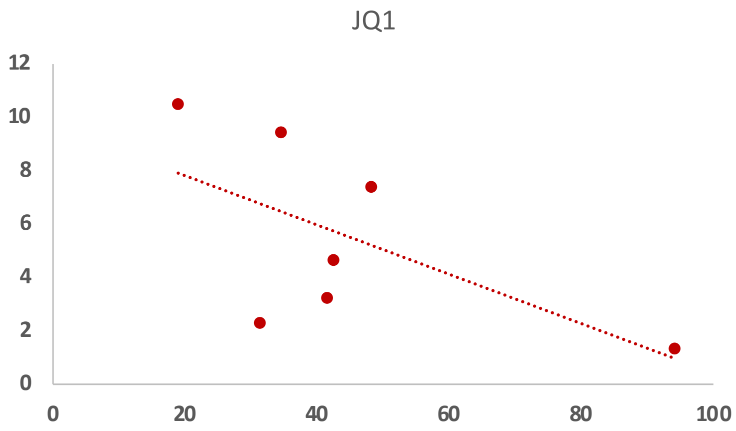
JQ1
12
10
8
6
4
2
0
0
20
40
60
80
100

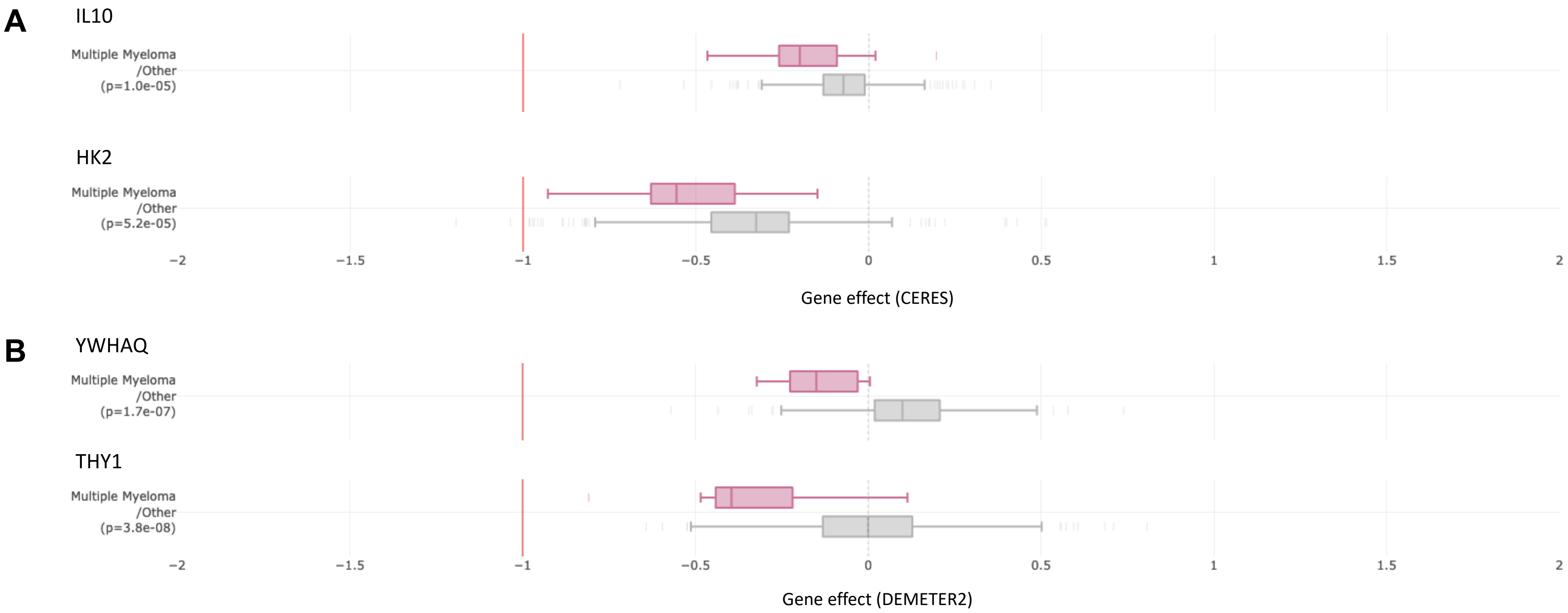

Supp Figure 11. Snapshots of gene loss-of-function screens from the Cancer Dependency Map project (https://depmap.org). (A) CRISPR/Cas9-based viability assays in MM cell lines (n = 20) and other cancer cell lines (n = 749). (B) RNAi-based viability assays in MM cell lines (n = 12) and other cancer cell lines (n = 534). DEMETER2 and CERES scores were calculated from RNAi-based and CRISPR/Cas9 viability screens, respectively. A lower score means that a gene is more likely to be dependent in a given cell line. A score of 0 is equivalent to a gene that is not essential whereas a score of -1 corresponds to the median of all commun essential genes. Boxplots in pink represent multiple myeloma cell lines and boxplots in grey correspond to other cancer cell lines.

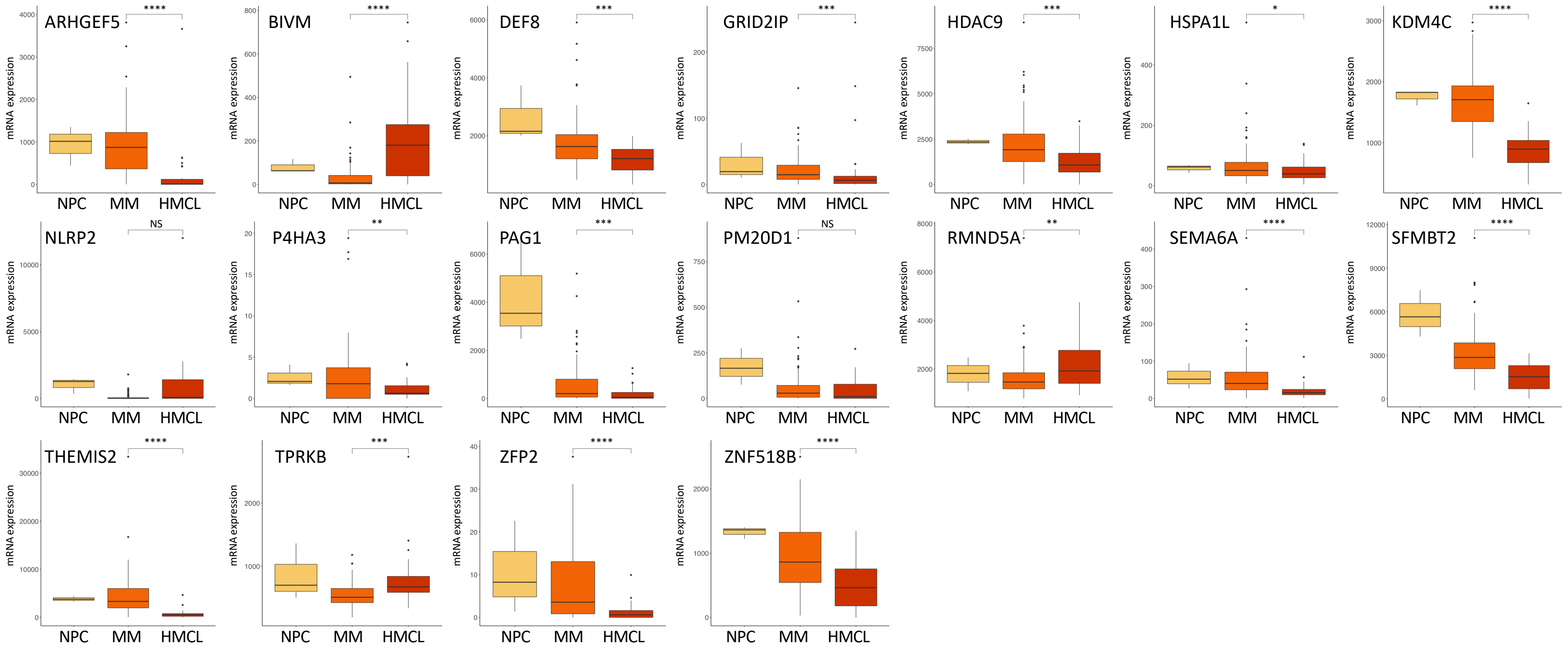

Supp Figure 12. Boxplots representing mRNA expression of genes composing repressive domain score in normal plasma cells (NPC; n = 3), plasma cells of MM patients (n = 97) and HMCLs (n = 33). Wilcoxon test. NS: non significant, **P*-value < 0.05, ***P*-value < 0.01, ****P*-value < 0.001, *****P*-value < 0.0001.

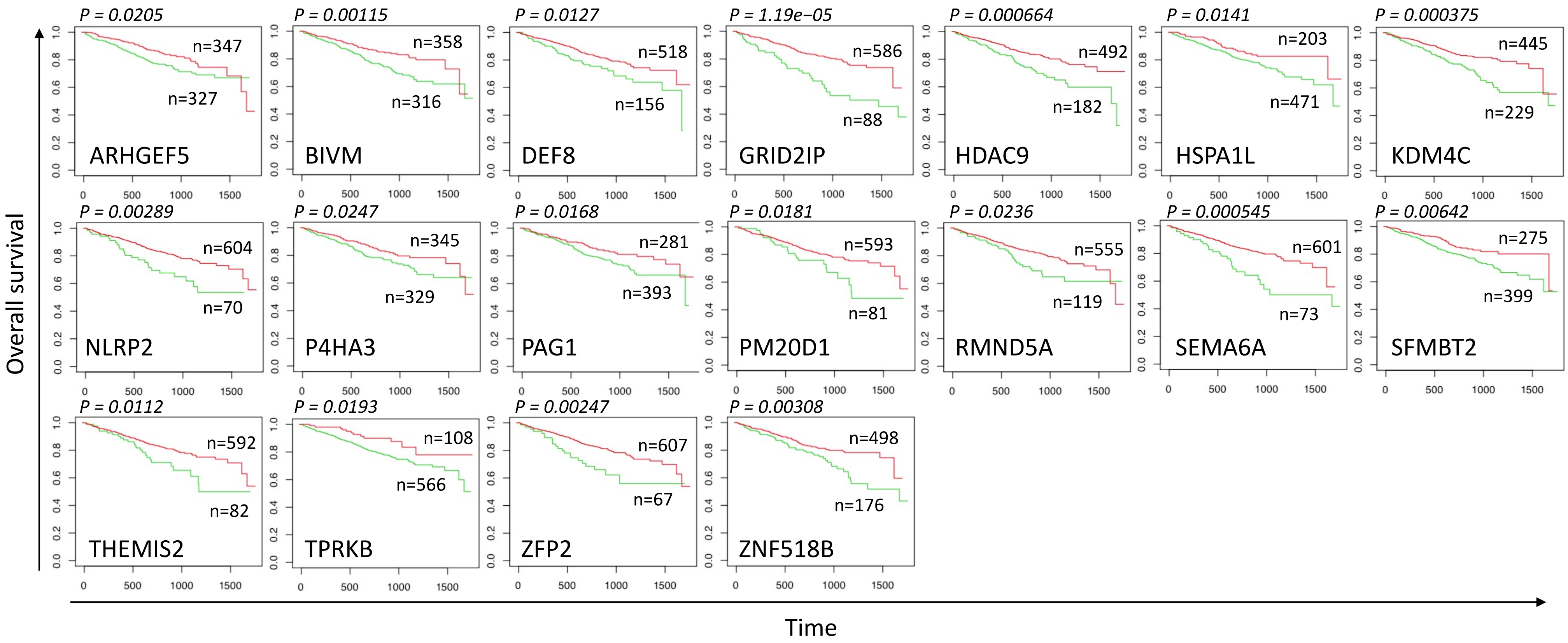

Supp Figure 13. Kaplan-Meier curves of genes composing repressive domain score in CoMMpass cohort (n = 674). Red curves represent low-risk group associated with high gene expression and green curves correspond to high-risk group associated with low gene expression

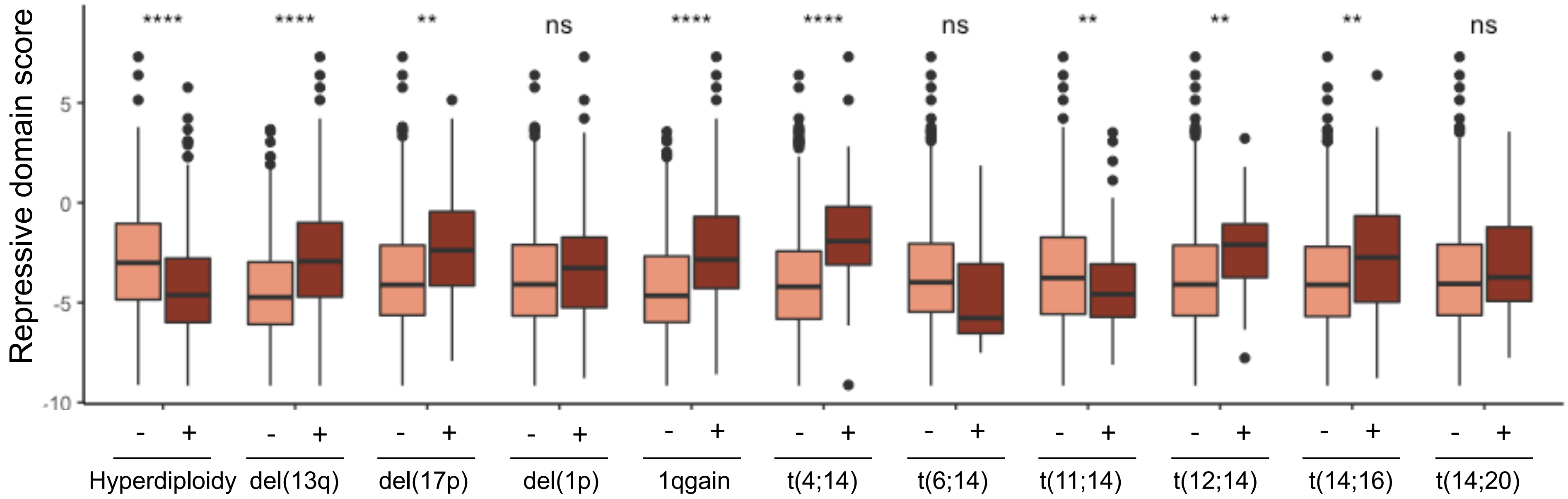

Supp Figure 14. Repressive domain score in cytogenetic abnormality subgroups of the CoMMpass cohort. Cytogenetic abnormality subgroups were composed of hyperdyploid (without: n=240, with: n=331), del(13p) (without: n=307, with: n=266), del(17p) (without: n=523, with: n=50), del(1p) (without: n=455, with: n=118), 1q gain (without: n=378, with: n=195), groups of patients and patients harboring t(4;14) (without: n=515, with: n=73), t(6;14) (without: n=572, with: n=16), t(11;14) (without: n=461, with: n=127), t(12;14) (without: n=564, with: n=24), t(14;16) (without: n=532, with: n=56), t(14;20) (without: n=543, with: n=45) translocations. Wilcoxon test. ns: non significant, **P*-value < 0.05, ***P*-value < 0.01, ****P*-value < 0.001, *****P*-value < 0.0001.

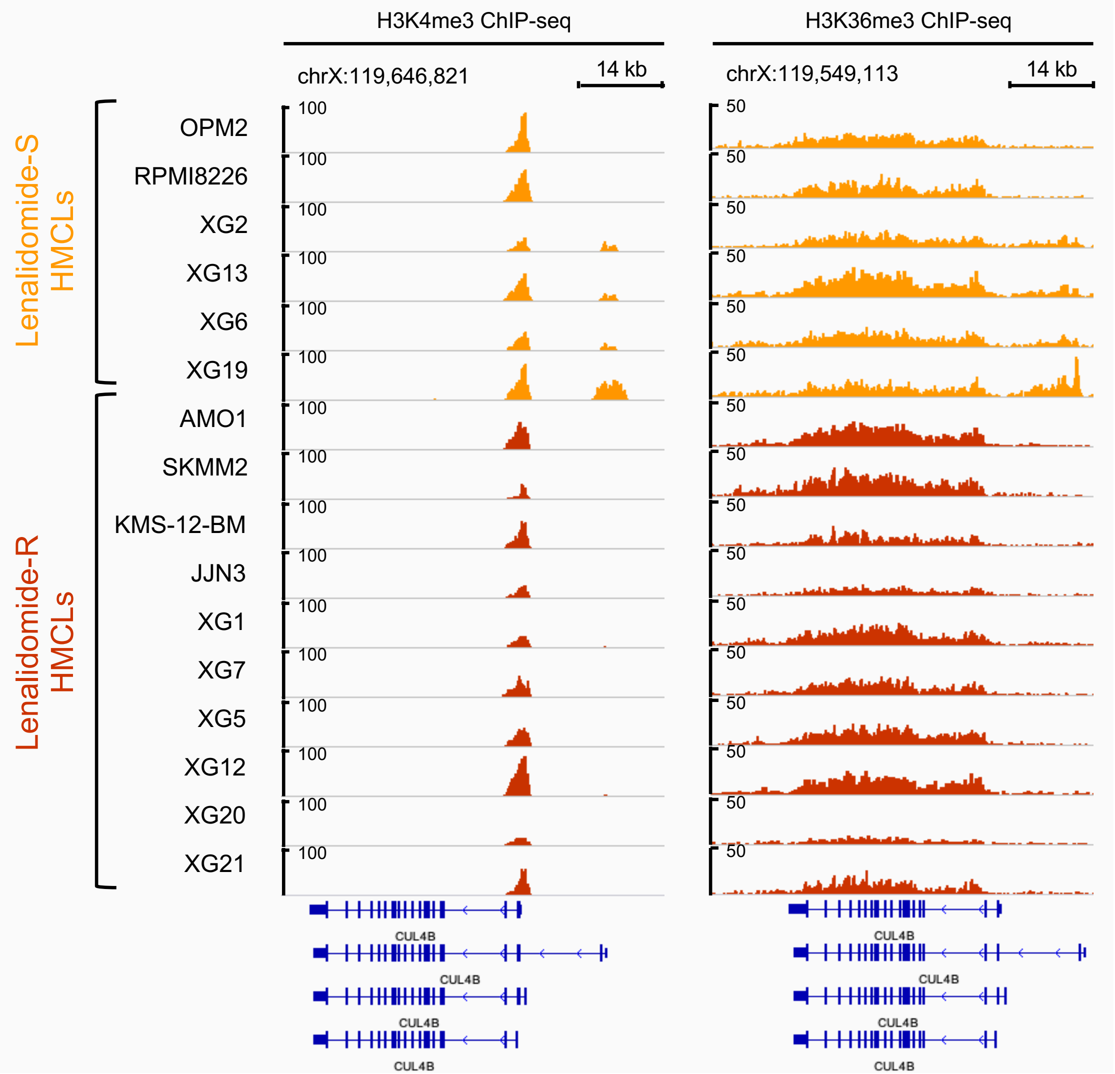

Supp Figure 15. *CUL4B* gene tracks of H3K4me3 and H3K36me3 ChIP-seq occupancy in lenalidomide-resistant and –sensitive HMCLs. 4/6 sensitive HMCLs seem to express transcript variant 1 (NM_003588.3) whereas 10/10 resistant HMCLs seem to express transcript variant 2 (NM_001079872.2). The x axis shows the genomic position and the y axis shows signal coverage of ChIP-seq occupancy in units of reads per bin mapped reads.

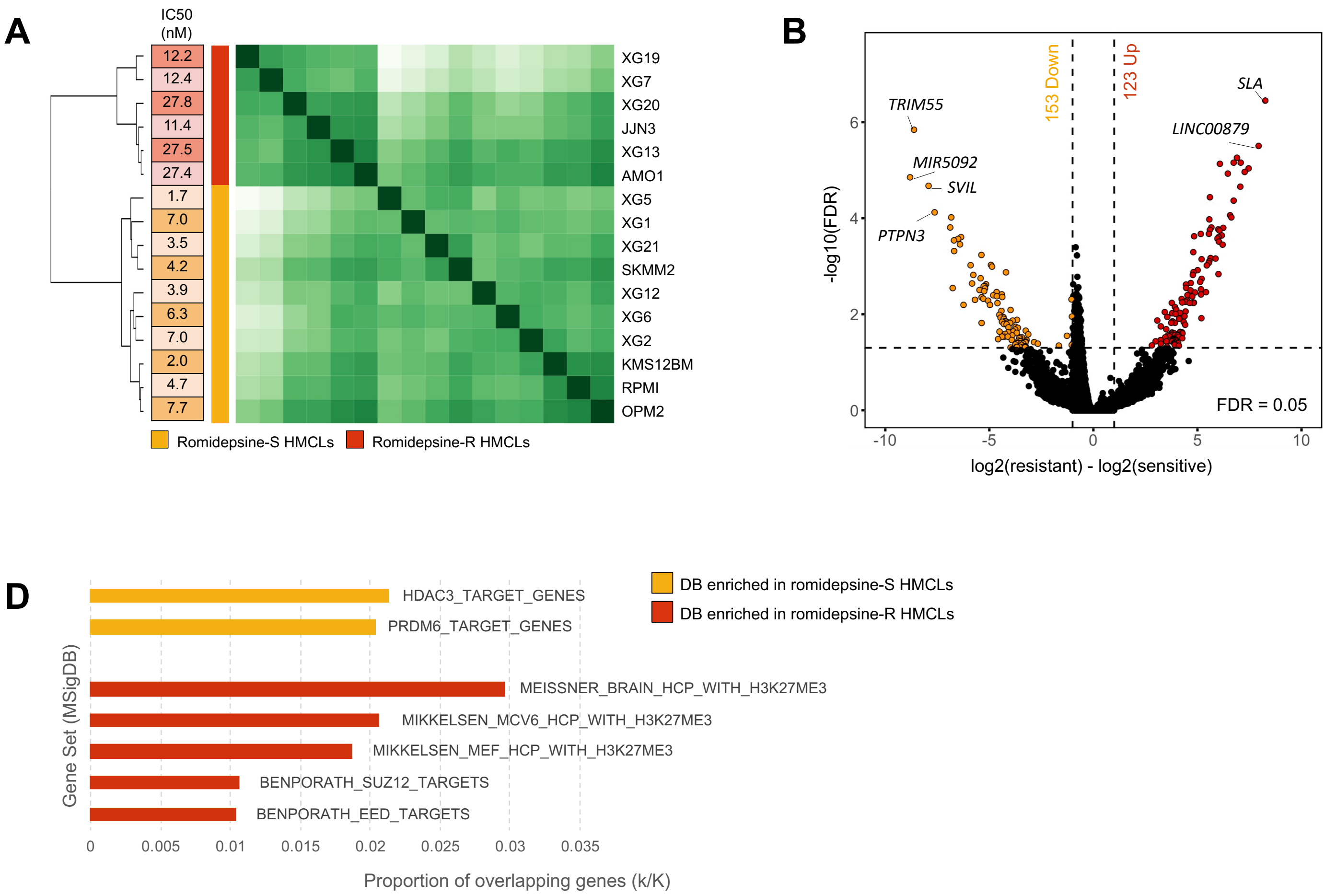

Supp Figure 16.

Supp Figure 16. H3K4me3 modification differentially enriched in romidepsin (ROMI) -sensitive and -resistant HMCLs. (A) Correlation heatmap using only the 2685 sites identified as being significantly differentially bound in romidepsin-resistant compared to romidepsin-sensitive HMCLs (FDR ≤ 0.05). (B) Volcano plot of differentially bound sites localized on promoters using romidepsin-resistant vs romidepsin-sensitive HMCLs contrast. Sites identified as significantly differentially bound in romidepsin-sensitive and -resistant HMCLs are colored in orange and red, respectively. (C) Gene tracks of H3K4me3 ChIP-seq occupancy. The promoter of *AFAP1-AS1* and *SILC1* genes is the most significantly differentially enriched by H3K4me3 modifications in romidepsin-sensitive group compared to romidepsin-resistant group and, conversely, the promoter of *FN3K* and *SLA* genes is the most significantly differentially enriched by H3K4me3 modifications in romidepsin-resistant group compared to romidepsin-sensitive group. The x axis shows the genomic position and the y axis shows signal coverage of ChIP-seq occupancy in units of reads per bin mapped reads. (D) Molecular signature of differentially bound sites localized on gene promoters enriched in romidepsin-sensitive and -resistant HMCLs was investigated using GSEA database (FDR ≤ 0.05).

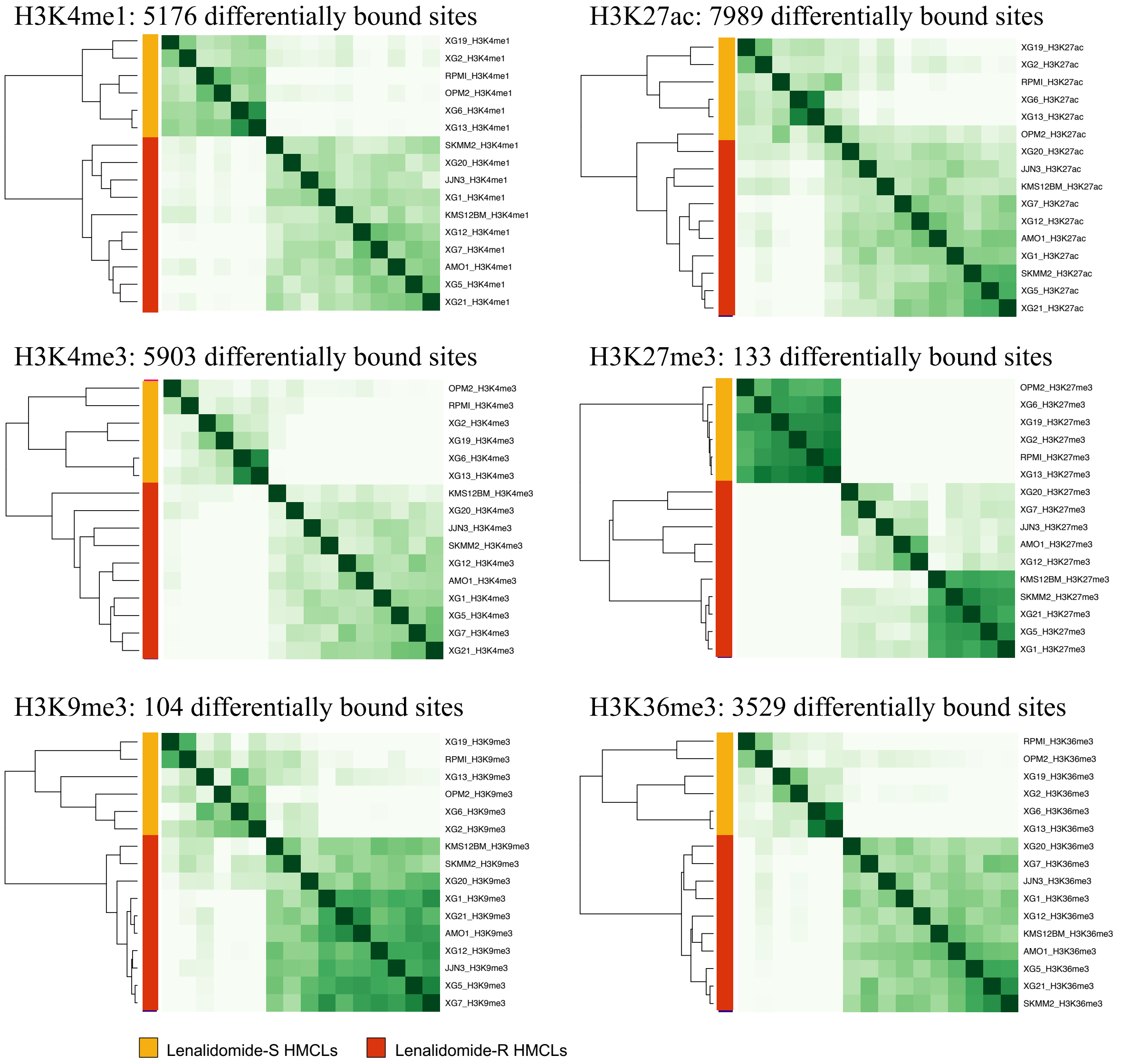

Supp Figure 17.The differentially bound sites identified using H3K4me1, H3K4me3, H3K9me3, H3K27ac, H3K27me3 and H3K36me3 in lenalidomide-resistant (R) and –sensitive (S) HMCLs.

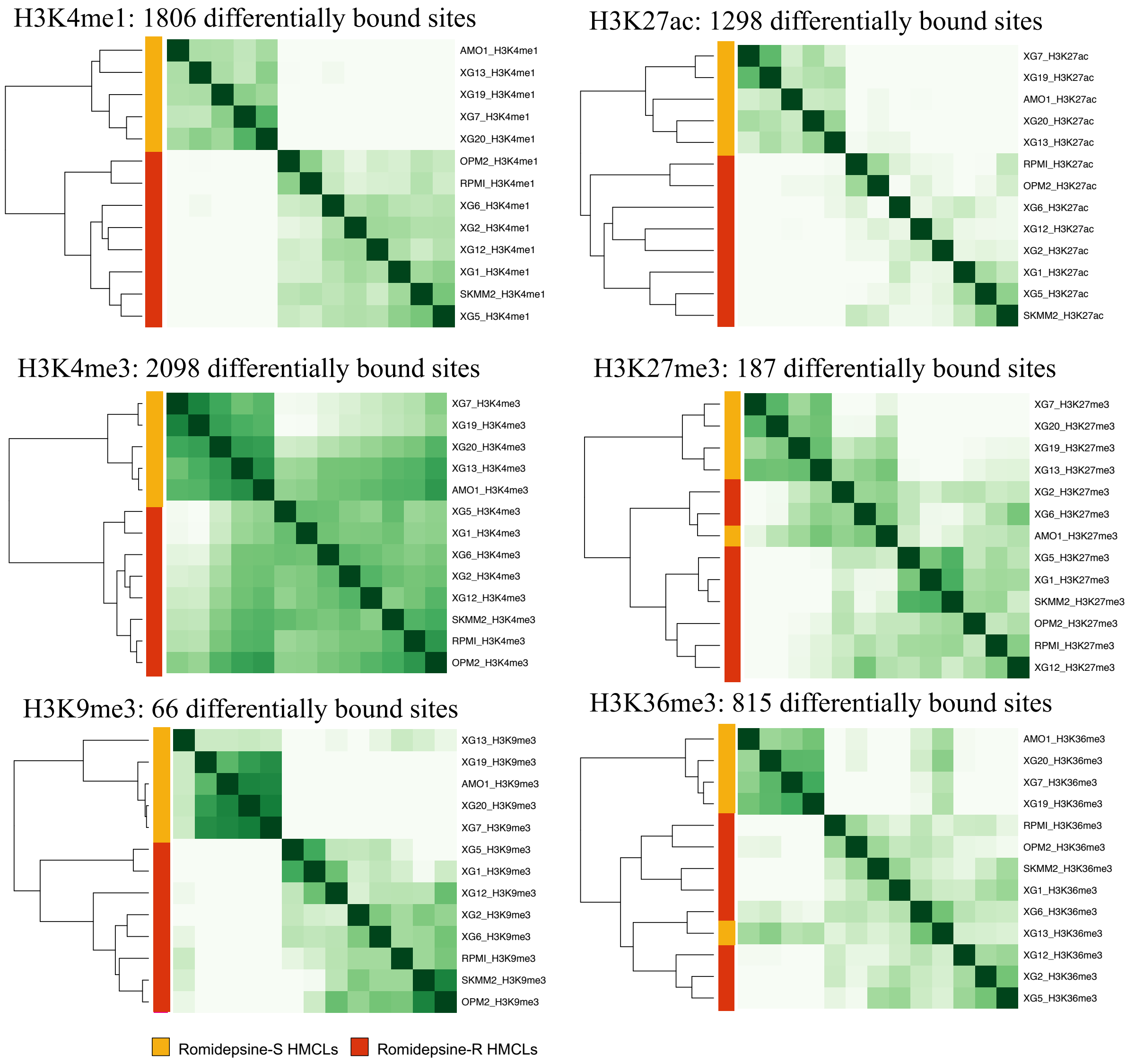

Supp Figure 18.The differentially bound sites identified using H3K4me1, H3K4me3, H3K9me3, H3K27ac, H3K27me3 and H3K36me3 in romidepsin-resistant (R) and –sensitive (S) HMCLs.
